# GPR35 agonists inhibit TRPA1-mediated colonic nociception through suppression of substance P release

**DOI:** 10.1097/j.pain.0000000000003399

**Published:** 2024-10-03

**Authors:** Rohit A. Gupta, James P. Higham, Abigail Pearce, Paulina Urriola-Muñoz, Katie H. Barker, Luke Paine, Joshua Ghooraroo, Tim Raine, James R. F. Hockley, Taufiq Rahman, Ewan St John Smith, Alastair J. H. Brown, Graham Ladds, Rie Suzuki, David C. Bulmer

**Affiliations:** a Department of Pharmacology, University of Cambridge, Tennis Court Road, Cambridge, United Kingdom; b Department of Gastroenterology, Addenbrookes Hospital, Cambridge University Teaching Hospitals, Cambridge, United Kingdom; c Nxera, Steinmetz Building, Granta Park Great Abington, Cambridge, United Kingdom

**Keywords:** Abdominal pain, TRPA1, GPR35, Substance P, Colonic nociception, Visceral hypersensitivity

## Abstract

The development of nonopioid analgesics for the treatment of abdominal pain is a pressing clinical problem. To address this, we examined the expression of G_i/o_-coupled receptors, which typically inhibit nociceptor activation, in colonic sensory neurons. This led to the identification of the orphan receptor GPR35 as a visceral analgesic drug target because of its marked coexpression with transient receptor potential ankyrin 1 (TRPA1), a mediator of noxious mechanotransduction in the bowel. Building on in silico docking simulations, we confirmed that the mast cell stabiliser, cromolyn (CS), and phosphodiesterase inhibitor, zaprinast, are agonists at mouse GPR35, promoting the activation of different G_i/o_ subunits. Pretreatment with either CS or zaprinast significantly attenuated TRPA1-mediated colonic nociceptor activation and prevented TRPA1-mediated mechanosensitisation. These effects were lost in tissue from GPR35^−/−^ mice and were shown to be mediated by inhibition of TRPA1-evoked substance P (SP) release. This observation highlights the pronociceptive effect of SP and its contribution to TRPA1-mediated colonic nociceptor activation and sensitisation. Consistent with this mechanism of action, we confirmed that TRPA1-mediated colonic contractions evoked by SP release were abolished by CS pretreatment in a GPR35-dependent manner. Our data demonstrate that GPR35 agonists prevent the activation and sensitisation of colonic nociceptors through the inhibition of TRPA1-mediated SP release. These findings highlight the potential of GPR35 agonists to deliver nonopioid analgesia for the treatment of abdominal pain.

## Abstract 2

Supplemental Digital Content is Available in the Text.

Consistent with its marked expression in colonic nociceptors, GPR35 agonists inhibit transient receptor potential ankyrin 1–mediated colonic nociception highlighting their utility for the treatment of visceral pain.

## 1. Introduction

The development of nonopioid analgesics for the treatment of chronic abdominal pain is a pressing issue because of the risk of dependence and addiction associated with long-term opioid use,^7,67^ and gut-specific side effects, including constipation and gastrointestinal bleeding, associated with both opioid^14,19^ and nonsteroidal anti-inflammatory drugs.^60,66^ Given the high prevalence of nociplastic abdominal pain evoked by conditions such as irritable bowel syndrome (IBS) and the rising incidence of chronic pain because of inflammatory bowel disease (IBD), there is a considerable unmet clinical need to identify new viscerospecific analgesic drug targets to address the marked personal and socioeconomic burden associated with chronic abdominal pain.^28,53,71^

The development of visceral hypersensitivity in response to mediators released from the bowel of patients with IBS or IBD is a key pathophysiological event, in which visceral nociceptors become sensitised and, therefore, signal pain in response to previously innocuous distension or contraction of the bowel.^13,30^ Numerous mechanisms contribute to the development of visceral hypersensitivity, such as enhanced activity of ion channels which mediate mechanosensation in the bowel, such as transient receptor potential ankyrin 1 (TRPA1) channels.^12^ Moreover, mechanosensitive afferent excitability can also be increased by depolarisation of the resting membrane potential or reduced threshold for action potential firing through modulation of ion channels, such as the voltage-gated sodium channels Na_V_1.8^35^ and Na_V_1.9.^33^ The mediators responsible for visceral hypersensitivity are not fully understood, although mast cell–derived mediators,^3,4^ such as histamine,^1^ serotonin (5-HT),^41^ nerve growth factor and tryptase,^18^ as well as lipids such as 5-oxo-ETE,^9^ are implicated in IBS. Immune cell mediators, such as cytokines (eg, tumour necrosis factor alpha, TNFα) and proteinases (eg, neutrophil elastase), are implicated in IBD.^5,6^ These mediators and mechanisms, therefore, represent novel drug targets for the development of visceral analgesics. However, there is a risk of redundancy when targeting a single sensitising agent given the diverse array of pronociceptive mediators present in the inflamed bowel.

An alternative approach is to stimulate G_i/o_-coupled receptors, which have the potential to inhibit nociceptor activation irrespective of the noxious stimulus because of their downstream effector responses, which enhance potassium channel conductance, reduce the pronociceptive effects associated with cyclic adenosine monophosphate accumulation, and suppress neuropeptide release.^40,70^ G_i/o_-coupled receptors comprise a broad range of members stimulated by agonists with analgesic activity, such as opioids and cannabinoids. To identify putative analgesic approaches for the treatment of abdominal pain in gastrointestinal disease, we compared the coexpression of transcripts encoding G_i/o_-coupled receptors with TRPA1, a mediator of colonic nociceptor mechanosensation (and mechanical hypersensitivity), using our existing single cell RNA-sequencing data from colonic sensory neurons.^34^ This led to the identification of the orphan G-protein–coupled receptor, GPR35, as a novel nonopioid visceral analgesic drug target. Consequently, we evaluated the effect of GPR35 agonists on TRPA1-mediated colonic nociceptor activation, mechanical hypersensitivity, colonic contractility, and neuropeptide release. This work demonstrates an antinociceptive role for GPR35 agonists, such as cromolyn (CS), and highlights their potential as visceral analgesics.

## 2. Methods

### 2.1. Ethical approval

Experiments using animal tissue were conducted in compliance with the Animals (Scientific Procedures) Act 1986 Amendment Regulations 2012 under Project Licences P7EBFC1B1 and PP5814995 granted to E. St. J. Smith by the Home Office with approval from the University of Cambridge Animal Welfare Ethical Review Body. Animals were euthanised by rising concentration of CO_2_ or cervical dislocation followed by exsanguination.

### 2.2. Animals

Adult male and female C57BL/6 mice (8-16 weeks) or male CD1 mice (8-16 weeks) were obtained from Charles River (Cambridge, United Kingdom; RRID:IMSR_JAX:000664) and male GPR35^+/+^ and GPR35^−/−^ mice (C57BL/6-Gpr35tm1b(EUCOMM)Hmgu/WtsiH) were rederived by MRC Harwell (Wellcome Trust Sanger Institute Mouse Genetics Project (Sanger MGP) and INFRAFRONTIER/EMMA (www.infrafrontier.eu)). Mice were housed in temperature-controlled rooms (21°C) with a 12-hour light/dark cycle and provided with nesting material, enrichment (eg, tubes, chewing blocks and shelters), and access to food and water ad libitum.

### 2.3. Molecular modelling and docking

A homology model of mouse GPR35 (mGPR35, Uniprot id: Q9ES90) was built using the published structure of human GPR35 receptor (hGPR35R, PDB id: 8H8J) using SwissModel (https://swissmodel.expasy.org/). The initial model of mGPR35 was then further refined through the GalaxyRefine 2 module of GalaxyWEB (https://galaxy.seoklab.org/). The refined mGPR35 model was used for docking alongside the published structure of hGPR35.

Docking of the chosen ligands was performed against the unliganded hGPR35 and modelled mGPR35 structures using published protocols.^15^ Briefly, the 3D structures of zaprinast (Zap), CS, and lodoxamide were obtained from PubChem and first blindly docked to the GPR35 structures using AutoDock 4.2.6. The best poses of the cromolyn and zaprinast drugs were then used for focused docking using GOLD suite version 5.3 (CCDC, Cambridge) using 10 independent docking runs. The 2D ligand interaction diagrams of the final docked poses were generated through PoseView implemented in the ProteinsPlus web server (https://proteins.plus/). All images were produced in PyMol version 1.3.

### 2.4. TRUPATH G protein dissociation assay

pRK5-HA-GRP35 was a gift from William Kaelin (Addgene plasmid # 204708; http://n2t.net/addgene:204708; RRID:Addgene_204708). TRUPATH triple Gαi/o plasmids were a gift from Justin English (Addgene plasmid # 196061; http://n2t.net/addgene:196061; RRID:Addgene_196061). Morphine hydrochloride was dissolved at 10 mM in water, and forskolin was dissolved at 10 mM in dimethyl sulfoxide (DMSO).

HEK293T cells were grown in DMEM/F12 Glutamax, supplemented with 10% foetal bovine serum, and 1% antibiotic–antimycotic solution. Cells were cultured at 37°C in 5% CO_2_, in a humidified incubator.

G protein activation was measured using the TRUPATH biosensor platform. HEK293T cells were transfected with HA-GPR35 and the TRUPATH triple plasmids for Gαi/o subunits, using polyethylenimine. After 24 hours, cells were harvested and seeded on a 0.01% poly-L-lysine–coated plate and grown for a further 24 hours. On the day of assay, media was removed, and cells washed with Hank Balanced Salt solution (HBSS). Cells were then incubated in HBSS containing 20 mM of HEPES, 1 mg/mL of bovine serum albumin (BSA), and 5 μM of coelenterazine 400a (NanoLight technologies) for 5 minutes, before the addition of ligands. Plates were read using a PheraStar microplate reader using the BRET2 module, at 60-second intervals. The BRET2 ratio was calculated (λ_515_/λ_400_) and total dissociation over a 30-minute period used to generate dose–response curves. pEC_50_ values were calculated using the “3 parameter log(agonist) vs response” model in GraphPad Prism9.

### 2.5. Ex vivo whole nerve electrophysiology

#### 2.5.1. Electrophysiological recording

Electrophysiological recordings of lumbar splanchnic nerve (LSN) activity were conducted as previously described.^11,52^ Briefly, the distal colorectum (splenic flexure to rectum) and associated LSN (rostral to inferior mesenteric ganglia) were isolated from mice euthanised as described above. The colon was cannulated and secured with fine thread (Gutermann) in a rectangular Sylgard-coated recording chamber (Dow Corning, Midland, MI). Tissue was bath superfused (7 mL/minute; 32-34°C) and luminally perfused (100 μL/min) by a syringe pump (Harvard apparatus, Holliston, MA) against a 2 to 3 mm Hg end-pressure with carbogenated Krebs buffer solution (95% O_2_, 5% CO_2_). Krebs buffer (in mM: 124 NaCl, 4.8 KCl, 1.3 NaH_2_PO_4_, 2.5 CaCl_2_, 1.2 MgSO_4_, 11.1 D-glucose and 25 NaHCO_3_) was supplemented with 10 μM of atropine and 10 μM of nifedipine to prevent smooth muscle activity.

Borosilicate suction electrodes were used to record the multi-unit activity of LSN bundles. Signals were amplified (gain 5 kHz), band pass filtered (100-1300 Hz; Neurolog, Digitimer Ltd, Welwyn, United Kingdom), and digitally filtered for 50-Hz noise (Humbug, Digitimer Ltd, Welwyn, United Kingdom). Analogue signals were digitized at 20 kHz (Micro1401; Cambridge Electronic Design, Cambridge, United Kingdom), and signals were visualised with Spike2 (Cambridge Electronic Design). Preparations underwent a minimum 30-minute stabilisation period before experiments began conducted to ensure baseline firing was stable.

Pilot studies examining the effect of the TRPA1 agonist ASP7663 on colonic afferent activity used tissue from CD1 mice. These studies examined the effect of a repeated 20-mL bath perfusion with 100 μM of ASP7663 (final bath concentration, 30 μM; hereafter, all concentrations refer to final bath concentration) at 45-minute intervals confirming marked tachyphylaxis to repeat application. Furthermore, separate experiments examined the concentration dependence of the ASP7663 response using 20-mL bath perfusion of ASP7663 at 30 μM, 9 μM, and 3 μM. The contribution of TRPA1 to ASP7663 responses was confirmed by bath perfusion with 20 mL of ASP7663 (9 μM) in the presence of the TRPA1 antagonist AM0902 (1 μM), with ASP7663 applied immediately after pretreatment with 100 mL AM0902.

Further studies were performed using tissue from wildtype C57BL/6 mice and littermate GPR35^+/+^ and GPR35^−/−^ mice. The effect of pretreatment with either 100 mL of vehicle (0.1% DMSO), 100 mL of CS (1 μM, 10 μM, or 100 μM) or 50 mL of Zap (10 μM or 100 μM) on the colonic afferent response to bath perfusion of 20 mL of ASP7663 (30 μM) was examined in tissue from GPR35^+/+^ mice. The effect of pretreatment with either 100 mL of vehicle (0.1% DMSO), 100 mL of CS (100 μM), or 50 mL of Zap (100 μM) was also examined on the colonic afferent response to bath perfusion with 20 mL of ASP7663 (30 μM) given in the presence of respective vehicle, CS, or Zap concentrations in tissue from GPR35^−/−^ mice. Experimenters were blinded to the application of vehicle or CS but not Zap because of the colour of the compound in solution.

In studies examining the effect of mast cell degranulation on colonic afferent firing, the response to bath perfusion with 100 mL compound 48/80 (50 µg/mL) was examined in tissue from wildtype C57BL/6 mice. Studies examining the effect of phosphodiesterase (PDE) inhibition on the colonic afferent response to ASP7663 measured the effect of pretreatment with either 100 mL of the pan PDE inhibitor IBMX (50 μM) or 100 mL of the PDE5/6 selective inhibitor sildenafil (1 μM) on the colonic afferent response to 20 mL of ASP7663 (30 μM) given in the presence of each PDE inhibitor.

In studies examining the afferent response to luminal distension and its sensitisation by ASP7663, repeated ramp distensions (0-80 mm Hg) were performed by occluding the luminal perfusion outflow (6 mL per hour), which gradually increased intraluminal pressure up to 80 mm Hg over approximately 1 to 2 minutes. When the maximum 80 mm Hg pressure was achieved, the luminal outflow was reopened, allowing pressure return to baseline. Pilot studies examined the effect of repeat luminal distension applied at 10-minute intervals, confirming that, although a modest reduction in the colonic afferent response to distension can be observed between the first and second repeat distension, the afferent response to the second and third repeat distension was comparable. As such, the effect of pretreatment with vehicle (0.1% DMSO, 100 mL), AM0902 (3 μM, 100 mL), CS (100 μM, 100 mL), and Zap (100 μM, 50 mL) on the afferent response to the third distension was examined. For studies investigating the effect of Zap and CS on the sensitisation of colonic afferent responses to luminal distension by ASP7663, pretreatment with either 50 mL of Zap (10 and 100 μM), 100 mL of CS (1, 10, and 100 μM), or respective 50 mL or 100 mL of vehicle (0.1% DMSO) began immediately after the second ramp distension, after which the bath was perfused with 20 mL of ASP7663 (30 μM) in the presence of respective pretreatments and the third luminal distension applied 10 minutes later. This experimental protocol was repeated in tissue from GPR35^−/−^ mice for 100 mL of vehicle (0.1% DMSO) and 100 mL of CS (100 μM) pretreatment. The experimenter was blinded vehicle and CS treatment but not Zap treatment because of the colour of the compound in solution.

For studies probing the contribution of substance P (SP) to colonic afferent firing and the response to ASP7663, we first examined the effect of 20 mL perfusion of ASP7663 (30 μM) alone, and in the presence of the neurokinin 1 receptor (NK_1_R) antagonist, aprepitant (10 μM), after a 100 mL pretreatment with aprepitant (10 μM). After this, we studied in separate experiments the effect of bath perfusion of 20 mL of SP at 3 μM, 9 μM, and 15 μM. In addition, we examined the effect of bath perfusion of 20 mL of SP (15 μM) in the presence of thiorphan (10 µM, a neprilysin/neutral endopeptidase inhibitor) and captopril (10 µM, an angiotensin converting enzyme inhibitor), after a 100 mL pretreatment with thiorphan (10 µM) and captopril (10 µM) alone and in the presence of aprepitant (10 µM). The concentrations of thiorphan and captopril used to prevent the breakdown of SP were based on previous work.^55^

We also examined the contribution of SP to the afferent response to distension (0-80 mm Hg) and its sensitisation by ASP7663. To do this, we examined the colonic afferent response to a third luminal distension applied after a 120 mL pretreatment with thiorphan (10 µM) and captopril (10 µM) alone, or after a 20 mL perfusion of SP (15 μM) in the presence of thiorphan (10 µM) and captopril (10 µM). In addition, we examined the colonic afferent response to a third luminal distension applied after a 100 mL pretreatment with either aprepitant (10 μM) or vehicle (0.1% DMSO) and a subsequent 20 mL application of ASP7663 (30 μM) given in the presence of respective pretreatments. Finally, the colonic afferent response to luminal distension was compared between a second and third luminal distension separated by a 100 mL perfusion with aprepitant alone (10 μM). The experimenter was blinded to treatment with vehicle or aprepitant.

Finally, we investigated the afferent response to prolonged, high-pressure distension of the colon by raising luminal pressure from 0 to 120 mm Hg over approximately 6 minutes (luminal perfusion of 1 mL per hour). This protocol was repeated in the presence of vehicle (0.1% DMSO), aprepitant (10 µM), and CS (100 µM). Pretreatments were applied after a second luminal distension to 40 mm Hg (luminal perfusion of 1 mL per hour) and continued throughout the distension to 120 mm Hg.

#### 2.5.2. Analysis

Lumbar splanchnic nerve discharge was determined by quantifying the number of spikes passing a manually determined threshold typically set at twice the background noise (60-80 µV) and binned to determine the firing rate (spikes/s). The change in firing rate was found by subtracting baseline activity (average firing rate in the 3 minutes preceding the stimulus) from nerve activity after stimulus application. To quantify mechanical hypersensitivity after drug application, the change in afferent firing during ramp 3 (postdrug) was compared with the change in afferent firing during ramp 2 (predrug) for individual treatments. To compare mechanical hypersensitivity, or lack thereof, between vehicle and treatment groups, postdrug responses (ramp 3) were normalised to predrug responses (ramp 2). As such, a peak percentage change in afferent firing during ramp 3 of ∼100% indicates no change in distension-evoked firing after treatment and, hence, no induction of mechanical hypersensitivity.

### 2.6. Ca^2+^ imaging

#### 2.6.1. Primary culture of dorsal root ganglia neurons

Cells from the DRG were cultured as described previously.^5^ In brief, the spine was removed and bifurcated to allow isolation of DRG. Dorsal root ganglia innervating the distal colon (T12-L5) were removed into ice-cold Leibovitz's L-15 GlutaMAX medium (supplemented with 2.6% (vol/vol) NaHCO_3_). Dorsal root ganglia were then incubated with 1 mg/mL of collagenase (15-minutes) followed by 1 mg/mL of trypsin (30-minutes), both with 6 mg/mL of bovine serum albumin (BSA) in Leibovitz's L-15 GlutaMAX medium (supplemented with 2.6% (vol/vol) NaHCO_3_). After removal of the enzymes, DRG were resuspended in 2 mL of Leibovitz's L-15 GlutaMAX medium containing 10% (vol/vol) foetal bovine serum (FBS), 2.6% (v/v) NaHCO_3_, 1.5% (vol/vol) glucose, and 300 units/mL of penicillin and 0.3 mg/mL of streptomycin (P/S). Dorsal root ganglia were mechanically triturated using a P1000 of Gilson pipette and centrifuged (100*g*) for 30 seconds. The supernatant, containing dissociated DRG neurons, was collected in a separate tube, and the pellet resuspended in 2 mL of Leibovitz's L-15 GlutaMAX medium containing 10% (vol/vol) FBS, 2.6% (vol/vol) NaHCO_3_, 1.5% (vol/vol) glucose, and P/S. This process was repeated 5 times, after which the total supernatant was centrifuged (100*g*) for 5 minutes to pellet the dissociated DRG neurons. Cells were resuspended in 250 µL of Leibovitz's L-15 GlutaMAX medium containing 10% (vol/vol) FBS, 2.6% (vol/vol) NaHCO_3_, 1.5% (vol/vol) glucose, and P/S, and plated (50 µL per dish) onto 35-mm glass bottomed dishes coated with poly-D-lysine (MatTek, Ashland, MA) and laminin (Thermo Fisher: 23017015). Dishes were incubated for 3 hours (37°C, 5% CO_2_) to allow cell adhesion and subsequently flooded with Leibovitz's L-15 GlutaMAX medium containing 10% (vol/vol) FBS, 2.6% (vol/vol) NaHCO_3_, 1.5% (vol/vol) glucose, and P/S. Neurons were used for experiments after 16 to 24 hours in culture.

#### 2.6.2. Imaging

Extracellular bath solution (in mM: 140 NaCl, 4 KCl, 1 MgCl_2_, 2 CaCl_2_, 4 glucose, 10 HEPES) was prepared and adjusted to pH 7.4 (using NaOH) and an osmolality of 290 to 310 mOsm (using sucrose). Culture medium was aspirated from neuronal cultures, and cells were incubated (30 minutes) with 100 µL Fluo-4-AM (10 μM) diluted in bath solution (room temperature; shielded from light). Before imaging, Fluo-4-AM was removed, and dishes were washed with bath solution.

Dishes were mounted on the stage of an inverted microscope (Nikon Eclipse TE-2000S), and cells were visualised with brightfield illumination at 10x magnification. To ensure a rapid exchange of solutions during protocols, the tip of a flexible perfusion inflow tube (AutoMate Scientific, Berkeley, CA) attached to a valve-controlled, gravity-fed perfusion system (Warner Instruments, Holliston, MA) was placed beside the field of view. Cells were superfused with bath solution to establish a baseline.

Fluorescent images were captured with a charge-coupled device camera (Retiga Electro, Photometrics, Tucson, AZ) at 2.5 fps with 100 milliseconds exposure. Fluo-4 was excited by a 470-nm light source (Cairn Research, Faversham, United Kingdom). Emission at 520 nm was recorded with μManager. All protocols began with a 10-second baseline before drug superfusion. SP (100nM) and capsaicin (1 µM) were sequentially applied, separated by a 4-minute wash period with bath solution. Finally, cells were stimulated with 50 mM of KCl for 10 seconds to determine cell viability, identify neurons, and allow normalisation of fluorescence.

#### 2.6.3. Analysis

Image analysis was performed using ImageJ (NIH, Bethesda, MD).^32^ Regions of interest were manually traced around individual cells. Average pixel intensity across each region of interest was measured and analysed with custom-written scripts in RStudio (RStudio, Boston, MA) to compute the proportion of neurons stimulated by each drug application. Briefly, the background fluorescence was subtracted from each region of interest, and fluorescence intensity (F) was baseline-corrected and normalised to the maximum fluorescence elicited during the application of 50 mM of KCl (F_pos_). Maximal fluorescence in KCl was denoted as 1 F/F_pos_. Further analysis was confined to cells with a fluorescence increase ≥5% above the mean baseline before 50 mM of KCl application. Using manual quality control, neurons were deemed responsive to drug application if a fluorescence increase of 0.1 F/F_pos_ was observed after drug superfusion. Responses to drug application were discounted if the increase in fluorescence began before or after the period of drug superfusion. Recordings without a stable baseline were also excluded from analysis. The proportion of drug-responsive neurons (ie, proportion of KCl-sensitive neurons exhibiting >0.1 F/F_pos_ increase in fluorescence during drug application) was measured for each experiment.

### 2.7. Chemiluminescent immunoassay to measure substance P release

The distal colon from GPR35^+/+^ and GPR35^−/−^ mice was removed, cleared of faecal material, and bathed in oxygenated Krebs solution. The tissue was washed 3 times with Krebs buffer before the experiment to remove any adhering tissue debris or blood that may interfere with the experiment. A segment of the colon (∼2 cm in length) was transferred into filtered carbogenated Krebs buffer and equilibrated at 37°C for 15 minutes. Tissue segments were then incubated with ASP7663 (100 µM) for 15 minutes after preincubation with either vehicle (Krebs solution), CS (100 µM), or AM0902 (3 µM) for 15 minutes. The experimenter blinded to the pretreatment. All incubations were conducted in the presence of thiorphan and captopril to prevent the breakdown of SP. After this, the solution bathing the colon was removed and centrifuged (500*g* for 5-minutes), with the resulting supernatant used to measure SP with a commercially available competitive chemiluminescent immunoassay (CLIA) kit according to the manufacturer's instructions (Cloud-Clone Corp., Houston, TX, Cat. no. CCA393Mu). The SP concentration in each sample was found through the construction of standard curves, and SP concentration was normalised to tissue weight.

### 2.8. Colonic contractility assay

The distal colon (∼2 cm) was removed from wildtype and GPR35^−/−^ mice, cleared of faecal material, and bathed in ice-cold carbogenated Krebs buffer until use. The colon was mounted vertically in a tissue bath filled with carbogenated Krebs buffer maintained at 37°C, with one end of the colon attached to an isotonic transducer, which converted changes in colon length into voltages. Signals were displayed using LabChart (ADInstruments). Tissue equilibrated for 60 minutes (washed every 15 minutes) before starting experimental protocols.

To assess baseline tissue contractility, 100 µM of acetylcholine was applied. After washout, ASP7663 (100 µM) was applied after a 10-minute preincubation with either vehicle (Krebs buffer), AM0902 (3 µM), aprepitant (10 µM), or CS (100 µM). The experimenter blinded to the pretreatment. At the end of each protocol, tissues were washed, and 100 µM of acetylcholine was reapplied to ensure that tissue contractility was unaffected and the tissue had not been paralysed. The response to ASP7663 in each condition was normalised to the peak response evoked by the first application of acetylcholine.

### 2.9. Statistics

All datasets were scrutinized to ensure that they met the assumptions (eg, normality assessed using Shapiro–Wilk test) of parametric analyses and, where appropriate, rank-based nonparametric analyses were used. Statistical analysis was performed in Prism 10 (Graphpad Inc, La Jolla, CA). Details of statistical tests used in each experiment are in the relevant figure legends. Data are displayed as mean ± standard error. *P*-value cutoffs in figures are denoted by *P < 0.05, **P < 0.01, and ***P < 0.001.

## 3. Results

### 3.1. GPR35 is highly coexpressed with transient receptor potential ankyrin 1 in colonic sensory neurons

**Figure 1.**
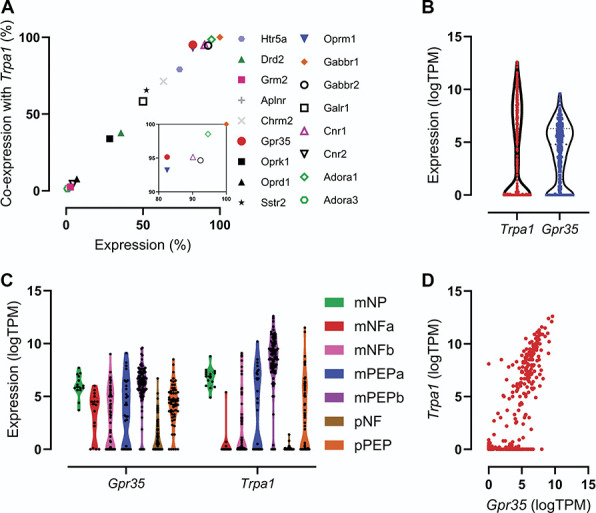
Expression of GPR35 in colonic sensory neurons. (A) Coexpression of transcripts encoding selected G_i/o_-coupled receptors with Trpa1 in colonic sensory neurons. Inset: enlargement of top-right cluster of data points. Data in (A-D) redrawn from Hockley et al., 2019.^34^ (B) Expression (log [transcripts per million]) of transcripts encoding Trpa1 and Gpr35 in colonic sensory neurons. (C) Expression of transcripts encoding Trpa1 and Gpr35 in each subpopulation of colonic sensory neuron. (D) Coexpression of transcripts encoding Trpa1 and Gpr35 in colonic sensory neurons. m, mixed lumbar splanchnic and pelvic afferents; NF, neurofilament-expressing; NP, nonpeptidergic; p, pelvic afferents; PEP, peptidergic.

To identify novel visceral analgesics, we examined the coexpression of G_i/o_-coupled receptors with TRPA1, an effector of noxious colonic mechanosensation, in colonic neurons using our previously published transcriptomic profiling of colon-innervating sensory neurons^34^ (Fig. 1A). Findings from this analysis identified a cluster of G_i/o_-coupled receptors highly coexpressed with TRPA1 in colonic neurons. These included the orphan receptor GPR35 (95.1% of *Trpa1*-positive neurons also expressed *Gpr35)* and receptors with known (or experimentally demonstrated) analgesic properties, such as the µ opioid receptor (*Oprm1*, 93.2%), the cannabinoid CB_1_ receptor (*Cnr1*, 95.1%), the adenosine A_1_ receptor (*Adora1*, 98.5%), and the GABA_B_ receptor (*Gabbr1*, 100%). GPR35 activation is not linked to central side effects, such as sedation, associated with opioid,^24^ cannabinoid,^36^ or GABA_B_ receptor agonists,^48^ or cardiovascular side effects associated with adenosine A_1_ receptor agonists.^50^ Both *Trpa1* and *Gpr35* transcripts were found to be highly expressed in colonic sensory neurons (Fig. 1B), with *Gpr35* broadly expressed across all colonic afferent populations, while *Trpa1* was more selectively expressed in peptidergic afferent populations (Fig. 1C). Given the high coexpression of *Gpr35* and *Trpa1* (Fig. 1D), we chose to further investigate the role of this receptor in the regulation of colonic mechanotransduction.

### 3.2. Cromolyn and zaprinast are agonists of GPR35

**Figure 2.**
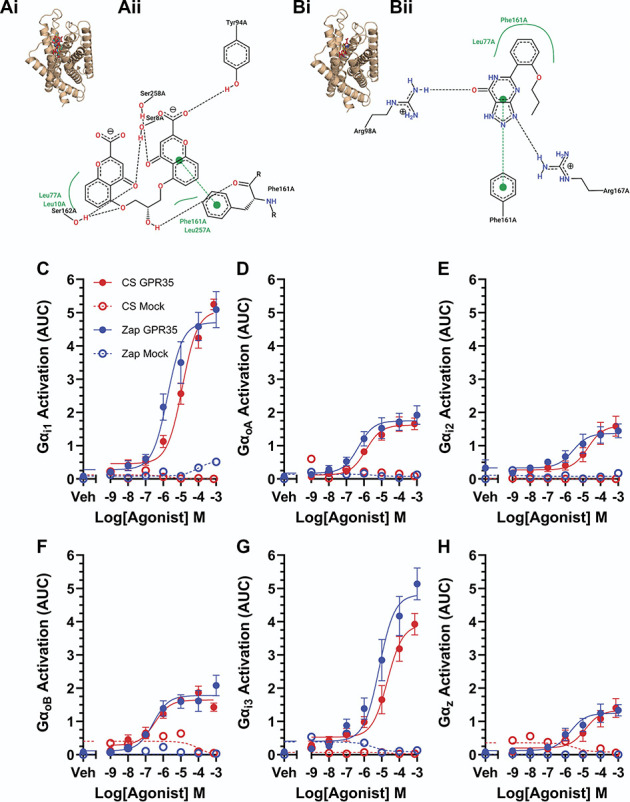
Cromolyn and zaprinast are agonists of GPR35. (A) (i) Predicted binding mode of cromolyn at mouse GPR35 based on the 10 best ranked binding poses obtained through independent docking runs in GOLD v.5.3. (ii) Ligand interaction diagram generated using PoseView. The most representative pose for cromolyn is shown. Dotted black lines indicate hydrogen bonding, solid green lines represent hydrophobic interactions, and dotted green lines represent π-π stacking. (B) (i) Predicted binding mode of zaprinast at mouse GPR35 based on the 10 best ranked binding poses obtained through independent docking runs in GOLD v.5.3. (ii) Ligand interaction diagram generated using PoseView. The most representative pose for zaprinast is shown. Dotted black lines indicate hydrogen bonding, solid green lines represent hydrophobic interactions, and dotted green lines represent π-π stacking. (C-H) Dissociation of given G_i/o_ subunits in response to stimulation of human GPR35 with CS (red) or Zap (blue). Filled symbols show results from cells transfected with GPR35; open symbols show mock-transfected cells. Points are plotted as mean ± SEM and fit with a three-parameter log [agonist]-response Boltzmann curve. CS, cromolyn.

We performed in silico semi-rigid blind docking followed by ligand pose refinement to predict the binding mode of 2 GPR35 agonists, CS,^38,39,69^ and Zap,^31,38,39,61^ at mouse GPR35 to determine their suitability for use in functional studies. Both CS (Fig. 2Ai) and Zap (Fig. 2Bi) appear to bind at the orthosteric site on mouse GPR35, where lodoxamide, a synthetic GPR35 agonist, is known to bind.^25^ There are clear differences in the predicted residues involved in the binding of CS and Zap to mouse GPR35, although π-π stacking with the phenyl group of Phe161 and a hydrophobic interaction with Leu77 appears to be important for the interaction of both ligands with the receptor (Figs. 2Aii and 2Bii).

Both CS and Zap were demonstrated to activate human GPR35 (expressed in HEK293T cells) using the TRUPATH biosensor platform for detecting G protein dissociation (Figs. 2C-H). Both ligands were able to activate all 6 G_i/o_ subunits tested with a similar potency. In mock-transfected cells lacking GPR35, negligible G protein dissociation was detected, indicating that the effects of CS and Zap were mediated by GPR35 activation and not by endogenously expressed G_i/o_-coupled receptors.

### 3.3. Transient receptor potential ankyrin 1–mediated colonic afferent activation is inhibited by stimulation of GPR35

After the identification of GPR35 as a putative visceral analgesic drug target, we sought to determine the ability of GPR35 agonists to prevent the activation of colonic afferents by the noxious TRPA1-selective agonist, ASP7663.^43^

Pilot studies conducted in tissue from CD1 mice confirmed that ASP7663 elicited a TRPA1-mediated increase in colonic afferent activity. These data showed that ASP7663 evoked a concentration-dependent increase in afferent firing of 0.6 ± 0.1 spikes/s at 3 µM (N = 5), 8.9 ± 0.5 spikes/s at 9 µM (N = 5), and 29.4 ± 7.0 spikes/s at 30 µM (N = 5), respectively (Supplemental Figure 1A, http://links.lww.com/PAIN/C131). The afferent response to 9 µM of ASP7663 was abolished by treatment with the TRPA1-selective antagonist, AM0902 (1 µM) (N = 5, *P* < 0.0001, Supplemental Figure 1B, http://links.lww.com/PAIN/C131). In addition, afferent responses to ASP7663 underwent marked tachyphylaxis on repeat application of ASP7663 at 30 µM (Supplemental Figure 1C and D, http://links.lww.com/PAIN/C131), limiting studies to a single application of ASP7663.

**Figure 3.**
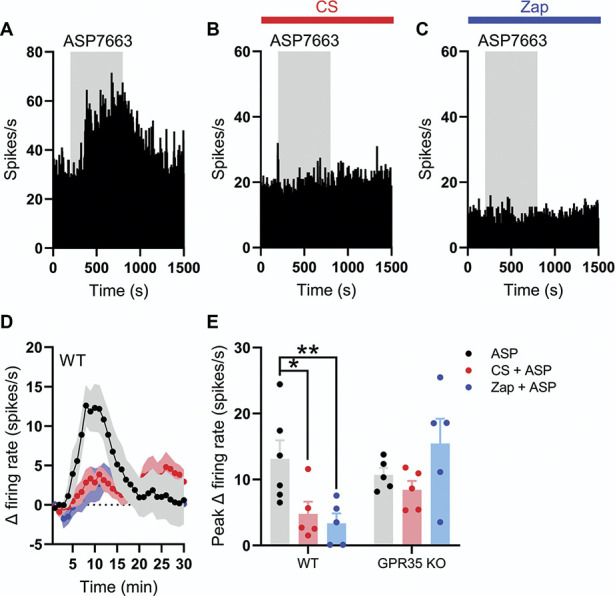
TRPA1-mediated colonic afferent firing is attenuated by activation of GPR35. (A) Example rate histogram showing the change in afferent firing evoked by the application of ASP7663 (grey shaded region). (B) Example rate histogram showing the change in afferent firing evoked by the application of ASP7663 (grey shaded region) in the presence of cromolyn (CS, 100 µM, red bar). (C) Example rate histogram showing the change in afferent firing evoked by the application of ASP7663 (grey shaded region) in the presence of zaprinast (Zap, 100 µM, blue bar). (D) Grouped data showing the mean change in afferent firing evoked by ASP7336 (points indicate the mean; shaded regions show the standard error) in the presence of either CS (100 µM, red) or Zap (100 µM, blue). (E) Grouped data showing the peak change in afferent firing evoked by ASP7336 in the presence of either CS (100 µM, red) or Zap (100 µM, blue) in both wildtype (WT) and GPR35^−/−^ animals. Data for each genotype analysed using a one-way ANOVA with Dunnett post-hoc tests. TRPA1, transient receptor potential ankyrin 1.

To test whether the activation of GPR35 could inhibit the response to ASP7663 (Fig. 3A), tissue from wild type mice was preincubated with increasing concentrations of CS (Fig. 3B) or Zap (Fig. 3C). Pretreatment with CS (1 µM) failed to attenuate ASP7663 (30 µM)-induced afferent firing (13.1 ± 2.8 spikes/s [N = 6] vs 9.9 ± 1.7 spikes/s [N = 5], *P* = 0.64). However, preincubation with 10 µM of CS did suppress afferent activity after ASP7663 application to 4.1 ± 1.3 spikes/s (*P* = 0.0065) and 100 µM of CS inhibited the afferent response to ASP7663 to a similar extent (4.8 ± 1.8 spikes/s, N = 5, *P* = 0.019, Figs. 3D and E). Preincubation with 10 µM of Zap did not affect ASP7663-induced afferent firing (12.0 ± 1.6 spikes/s, N = 5, *P* = 0.99), although treatment with 100 µM of Zap substantially reduced the effect of ASP7663 on afferent firing (3.4 ± 1.5 spikes/s, N = 5, *P* = 0.0051, Figs. 3D and E).

To verify that the effect of CS and Zap on ASP7663-evoked afferent discharge was mediated by GPR35, we repeated these experiments in tissue from GPR35^−/−^ animals. The afferent response to ASP7663 in tissue from GPR35^−/−^ mice was comparable to that in wild type (10.7 ± 1.0 spikes/s, N = 5). However, neither 100 µM of CS (8.4 ± 1.3 spikes/s, N = 5, *P* = 0.51) nor 100 µM of Zap (15.5 ± 3.7 spikes/s, N = 5, *P* = 0.33) had any effect on ASP7663-induced afferent firing (Fig. 3E).

Finally, we sought to understand any possible contribution of the additional pharmacological activity of CS, which is a mast cell stabiliser,^10^ and Zap, which also inhibits phosphodiesterase (PDE) activity.^46^ Consistent with previous reports in healthy colonic tissue,^20^ application of the mast cell degranulator, compound 48/80 (50 µg/mL), had no effect on colonic afferent nerve discharge indicating that mast cells were unlikely to have contributed to ASP7663-mediated nerve activation and, consequently, the inhibitory actions of CS (Supplemental Figure 2A, http://links.lww.com/PAIN/C131). Furthermore, neither IBMX (50 µM, nonselective PDE inhibitor) nor sildenafil (1 µM, selective PDE5/6 inhibitor) attenuated ASP7663-induced colonic afferent firing (Supplemental Figure 2B and C, http://links.lww.com/PAIN/C131), suggesting that the suppression of afferent activity by Zap was independent of PDE inhibition.

### 3.4. Inhibition of transient receptor potential ankyrin 1 but not GPR35 receptor activation attenuates colonic afferent mechanosensitivity

Having established that GPR35 agonists CS and Zap attenuate ASP7663-induced colonic afferent firing, we next sought to investigate the effect of GRP35 receptor activation on the afferent response to colorectal distension.

**Figure 4.**
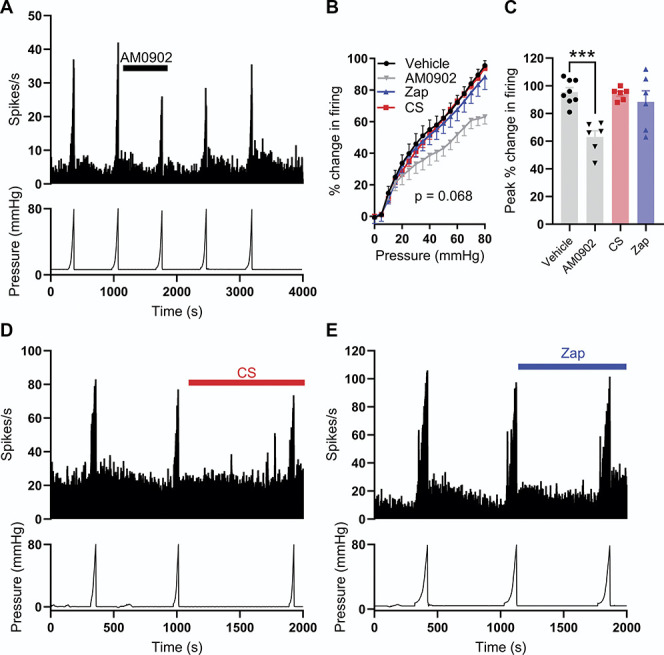
TRPA1 contributes to colonic afferent mechanosensitivity. (A) Example recording showing afferent firing during 5 successive ramp distensions with AM0902 (1 mM) applied before and during the third ramp distension. (B) Grouped data showing the percentage change in afferent firing during the third ramp distension in vehicle- (black), AM0902- (grey), CS- (red), and Zap- (blue) treated tissue. Two-way repeated measures ANOVA with Dunnett post-hoc tests. The percentage change in firing is relative to peak firing during the preceding ramp distension. (C) Grouped data showing the peak change in afferent firing during the third ramp distension in vehicle-, AM0902-, CS-, and Zap-treated tissue. One-way ANOVA with Dunnett post-hoc tests. (D) Example recording showing afferent firing during 3 consecutive ramp distensions with CS applied before and during the third ramp distension. (E) Example recording showing afferent firing during 3 consecutive ramp distensions with Zap applied before and during the third ramp distension. CS, cromolyn; TRPA1, transient receptor potential ankyrin 1.

Transient receptor potential ankyrin 1 has been shown to be an effector of colonic afferent mechanosensitivity, and, in keeping with these reports, we confirmed that pretreatment with a TRPA1-selective antagonist, AM0902 (1 µM), attenuated the colonic afferent response to colonic distension compared to vehicle-treated tissue (main effect of drug, F(3, 22) = 2.73, *P* = 0.068, Figs. 4A and B) at distension pressures >35 mm Hg (*P* < 0.038). There was a marked reduction in the peak afferent response to colorectal distension in tissues pretreated with AM0902 (N = 6) compared to vehicle (N = 8, F(3, 22) = 9.86, *P* = 0.0002, Figure 4C and Supplemental Figure 3A, http://links.lww.com/PAIN/C131). Application of AM0902 did not affect colonic compliance (Supplemental Figure 3B, http://links.lww.com/PAIN/C131).

By contrast, pretreatment with CS (100 µM, N = 6) did not affect the colonic afferent response to distension (main effect of drug, *P* = 0.068, Figs. 4B and D) at any intraluminal pressure (*P* > 0.81), and there was no difference in the peak afferent response to colonic distension between vehicle- and CS-treated tissues (*P* = 0.99, Fig. 4C). Zap (100 µM, N = 6) also had no effect on the colonic afferent response to ramp distension (main effect of drug, *P* = 0.068, Figs. 4B and E) at any intraluminal pressure (*P* > 0.58), and the peak afferent response to colonic distension was unaffected by Zap (*P* = 0.58, Fig. 4C). Neither the application of CS (Supplemental Figure 3C, http://links.lww.com/PAIN/C131) nor the application of Zap (Supplemental Figure 3D, http://links.lww.com/PAIN/C131) had any effect on colonic compliance.

### 3.5. Activation of GPR35 inhibits ASP7663-induced mechanical hypersensitivity

**Figure 5.**
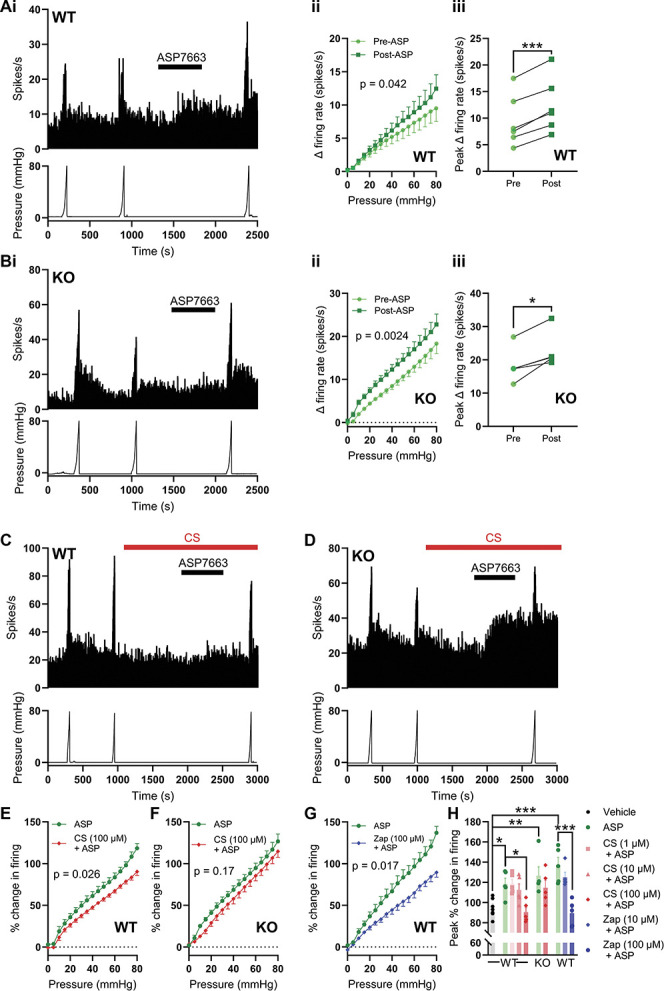
GPR35 activation inhibits TRPA1-induced mechanical hypersensitivity. (A) (i) Example recording showing the sensitisation of the afferent response to ramp distension of the colon by ASP7663 in tissue from a wildtype mouse. (ii) Grouped data showing the change in afferent firing during ramp distension of the colon before and after ASP7663 application. Two-way repeated measures ANOVA. (iii) Grouped data showing the peak change in afferent firing during ramp distension of the colon before and after ASP7663 application. Two-tailed ratio-paired *t* test. (B) (i) Example recording showing the sensitisation of the afferent response to ramp distension of the colon by ASP7663 in tissue from a GPR35^−/−^ mouse. (ii) Grouped data showing the change in afferent firing during ramp distension of the colon before and after ASP7663 application. Two-way repeated measures ANOVA. (iii) Grouped data showing the peak change in afferent firing during ramp distension of the colon before and after ASP7663 application. Two-tailed ratio-paired *t* test. (C) Example recording from tissue from a wildtype mouse showing the inhibition of ASP7663-induced afferent firing and sensitisation of the response to ramp distension by pretreatment with CS. (D) Example recording from tissue from a GPR35^−/−^ mouse showing the loss of the inhibitory effect of CS on ASP7663-induced afferent firing and sensitisation of the response to ramp distension. (E) Grouped data showing the change in afferent firing during the third (post-ASP7663) ramp distension in tissue from wildtype mice treated with ASP7663 with or without CS. Two-way repeated measures ANOVA with Holm–Sidak post-hoc tests. (F) Grouped data showing the change in afferent firing during the third (post-ASP7663) ramp distension in tissue from GPR35^−/−^ mice treated with ASP7663 with or without CS. Two-way repeated measures ANOVA with Holm–Sidak post-hoc tests. (G) Grouped data showing the change in afferent firing during the third (post-ASP7663) ramp distension in tissue from wildtype mice treated with ASP7663 with or without Zap. Two-way repeated measures ANOVA with Holm–Sidak post-hoc tests. (H) Grouped data showing the peak change in afferent firing during the third (postvehicle or -ASP7663) ramp distension. One-way ANOVA with Bonferroni post-hoc tests. CS, cromolyn; TRPA1, transient receptor potential ankyrin 1.

To explore the relationship between mechanosensitivity, TRPA1, and GPR35 receptor activation further, we investigated the effect of GPR35 agonist pretreatment on TRPA1 agonist–induced mechanical hypersensitivity. Application of ASP7663 (30 µM, Fig. 5Ai) augmented the afferent response to colonic distension (main effect of drug, F(1,5) = 7.38, *P* = 0.042; Figs. 5Ai and ii) resulting in an elevated peak increase in afferent firing during ramp distension (9.50 ± 2.0 spikes/s pre-ASP7663 vs 12.5 ± 2.1 spikes/s post-ASP7663, N = 6, *P* = 0.0001, Fig. 5Aiii). There was a significant interaction between drug and pressure (F(1.71, 8.53) = 11.5, *P* = 0.0047, Fig. 5Aii), which, in conjunction with the data in Figure 5Aiii, indicates that ASP7663 may sensitise high-threshold mechanosensitive afferents. Furthermore, the application of ASP7663 elicited a comparable mechanical hypersensitivity in tissue from GPR35^−/−^ mice (main effect of drug, F(1,4) = 47.0, *P* = 0.0024 Figs. 5Bi and ii), resulting in an increase in peak afferent firing during ramp distension from 18.3 ± 2.3 spikes/s to 22.8 ± 2.4 spikes/s (N = 5, *P* = 0.012, Fig. 5Biii). The proportional change in peak distension–evoked afferent firing induced by ASP7663 was no different between wildtype and GPR35^−/−^ tissue (*P* = 0.40). No change in colonic compliance was observed after the application of ASP7663 in tissue from wildtype or GPR35^−/−^ mice (Supplemental Figure 4A, http://links.lww.com/PAIN/C131).

Consistent with GPR35 agonist–mediated inhibition of ASP7663-evoked afferent firing, pretreatment with CS attenuated ASP7663-induced mechanical hypersensitivity in wildtype (Fig. 5C), but not GPR35^−/−^ (Fig. 5D), tissue. In wildtype tissue, preincubation with 100 µM of CS attenuated ASP7663-induced mechanical hypersensitivity (main effect of drug, F(1, 8) = 7.41, *P* = 0.026, Fig. 5E). However, 100 µM of CS failed to suppress ASP7663-induced mechanical hypersensitivity in tissue from GPR35^−/−^ animals (main effect of drug, F(1, 8) = 2.33, *P* = 0.17, Fig. 5F). Zap (100 µM) exhibited a similar effect to that of CS and was found to inhibit ASP7663-induced mechanical hypersensitivity in wildtype tissue (main effect of drug, F(1, 8) = 9.02, *P* = 0.017, Fig. 5G).

The effect of CS and Zap on the peak change in firing evoked by colonic distension was concentration-dependent. Neither 1 µM (*P* > 0.99, N = 5) nor 10 µM (*P* > 0.99, N = 5) of CS inhibited the effect of ASP7663 on peak distension-evoked afferent activity (Fig. 5H), whereas 100 µM of CS did suppress the peak change in afferent activity (F(9, 43) = 7.90, *P* = 0.016, N = 5, Fig. 5H). In tissue from GPR35^−/−^ animals, 100 µM of CS did not reduce the peak change in distension-evoked afferent firing after ASP7663 application (*P* > 0.99, N = 5, Fig. 5H). Finally, while 10 µM of Zap was without effect (*P* > 0.99), 100 µM of Zap suppressed peak distension–evoked afferent activity after ASP7663 application (*P* < 0.0001, N = 5, Fig. 5H). Peak distension–evoked firing in each group treated with ASP7663 alone was greater than that in a vehicle-treated group (N = 8), verifying ASP7663-induced mechanical hypersensitivity (Fig. 5H). The application of CS (Supplemental Figure 4B, http://links.lww.com/PAIN/C131) or Zap (Supplemental Figure 4C, http://links.lww.com/PAIN/C131) with ASP7663 did not have any effect on colonic compliance.

### 3.6. Substance P contributes to transient receptor potential ankyrin 1–mediated colonic afferent activation by

We have thus shown that the GPR35 agonists, CS and Zap, attenuate TRPA1-induced afferent activation and mechanical hypersensitivity, but not the activation of colonic afferents by luminal distension alone. Therefore, we reasoned that GPR35 receptor activation might block release of neuropeptides from colonic afferent terminals in response to TRPA1 activation rather than having a direct inhibitory effect on the afferent terminal or TRPA1 channel activity, both of which would be expected to attenuate distension-evoked afferent responses. Agonist activation of TRPA1 has previously been shown to produce neurogenic inflammation through the release of SP from sensory neuron terminals,^27,49^ an effect blocked by pretreatment with agonists of G_i/o_-coupled receptors.^44^

**Figure 6.**
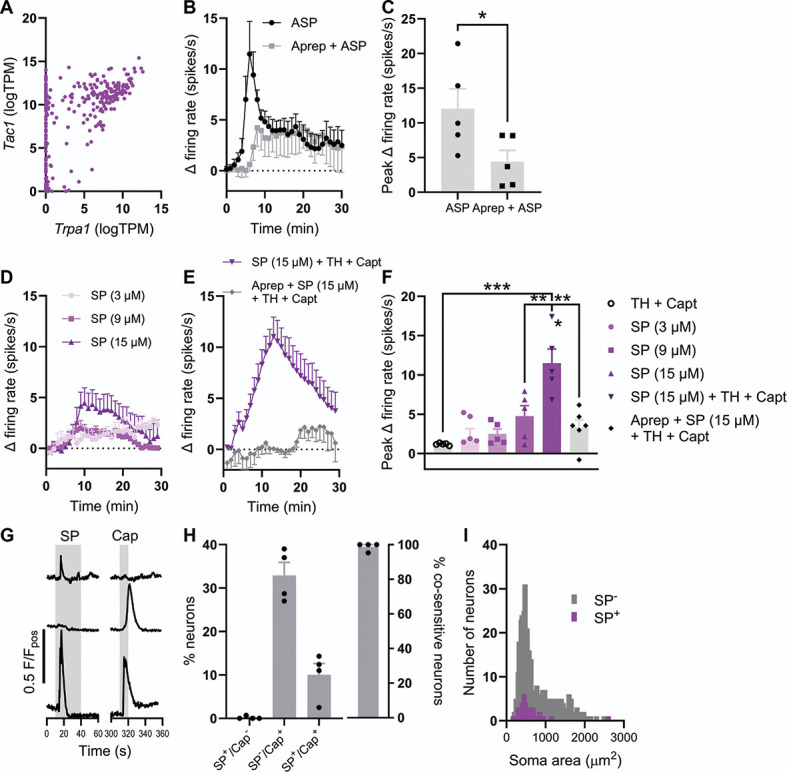
SP stimulates colonic afferents and cultured sensory neurons. (A) Coexpression of Trpa1 and Tac1 transcripts in colonic sensory neurons. Data redrawn from Hockley et al., 2019. (B) Grouped data showing the change in afferent firing rate after the application of ASP7663 (30 µM) alone or after tissue pretreatment with aprepitant (10 µM). (C) Grouped data showing the peak change in afferent firing rate after the application of ASP7663 (30 µM) alone or after tissue pretreatment with aprepitant (10 µM). Two-tailed unpaired *t* test. (D) Grouped data showing the change in afferent firing rate after the application of 3, 9, or 15 µM of SP. (E) Grouped data showing the change in afferent firing rate after the application of SP with thiorphan (TH) and captopril (Capt) in the absence and presence of aprepitant. (F) Grouped data showing the peak change in afferent firing rate from the experiments shown in (D and E). One-way ANOVA with Bonferroni post-hoc tests. (G) Example Fluo-4 fluorescence traces showing 3 distinct response profiles after the application of SP and capsaicin. (H) (Left) Grouped data showing the proportion of neurons which responded to SP alone, capsaicin alone, or to both SP and capsaicin. (Right) Grouped data showing the proportion of SP-sensitive neurons which were cosensitive to capsaicin. (I) Histogram showing the distribution of sensory neuron soma size for SP-sensitive (purple) and SP-insensitive (grey) neurons. Median soma areas compared using a Mann–Whitney *U* test. SP, substance P.

To investigate this, we first confirmed that the gene encoding SP, *Tac1*, is highly coexpressed with *Trpa1* in colonic sensory neurons (Fig. 6A). After this, we demonstrated that pretreatment with the NK_1_ receptor antagonist, aprepitant (10 µM), blunted the afferent response to ASP7663 (30 µM, Fig. 6B), reducing the peak firing elicited by ASP7663 from 12.1 ± 2.9 spikes/s (N = 5) to 4.4 ± 1.6 spikes/s (N = 5, *P* = 0.048, Fig. 6C), indicating that SP and NK_1_ receptor signalling contribute to the afferent response to TRPA1 activation.

Modest changes in colonic afferent activity were initially found in response to the application of 3 µM, 9 µM, and 15 µM of SP (Fig. 6D), resulting in a peak increase of 1.9 ± 1.3 spikes/s (N = 5), 2.5 ± 0.6 spikes/s (N = 5), and 4.8 ± 1.3 spikes/s (N = 5), respectively. However, a robust increase in colonic afferent activity in response to SP was observed after pretreatment of tissue with protease inhibitors, thiorphan (10 µM; neprilysin/neutral endopeptidase inhibitor) and captopril (10 µM; angiotensin converting enzyme inhibitor), highlighting the impact of proteolytic degradation on exogenously applied SP (Fig. 6E). The application of 15 µM of SP in the presence of thiorphan and captopril elicited a peak increase in afferent firing of 11.5 ± 1.8 spikes/s (N = 5, F(5, 26) = 10.1, *P* = 0.0035 compared to 15 µM of SP alone, Figs. 6E and F). The addition of aprepitant abrogated the peak afferent response to SP (3.4 ± 1.0 spikes/s, N = 5, *P* = 0.0002, Figs. 6E and F). The application of thiorphan and captopril alone did not stimulate any appreciable afferent firing (1.2 ± 0.1 spikes/s, N = 5, Fig. 6F).

Finally, we further confirmed the stimulatory effect of SP on sensory neurons in culture (where proteinases are not likely to be present), observing a rise in cytosolic [Ca^2+^] in 52 of 497 neurons (from 4 animals) after the application of 100 nM of SP. The majority (51 of 52) of SP-sensitive neurons were cosensitive to the TRPV1 agonist, capsaicin (1 µM, Figs. 5G and H), a key feature of a subset of nociceptive sensory neurons. Substance P-sensitive neurons were also found to be of a smaller soma size (median soma area: 491 µm^2^; interquartile range: 408-646 µm^2^) compared to SP-insensitive neurons (553 µm^2^, interquartile range: 423-956 µm^2^, *P* = 0.022, Fig. 6I).

### 3.7. ASP7663-induced mechanical hypersensitivity is dependent on substance P signalling

**Figure 7.**
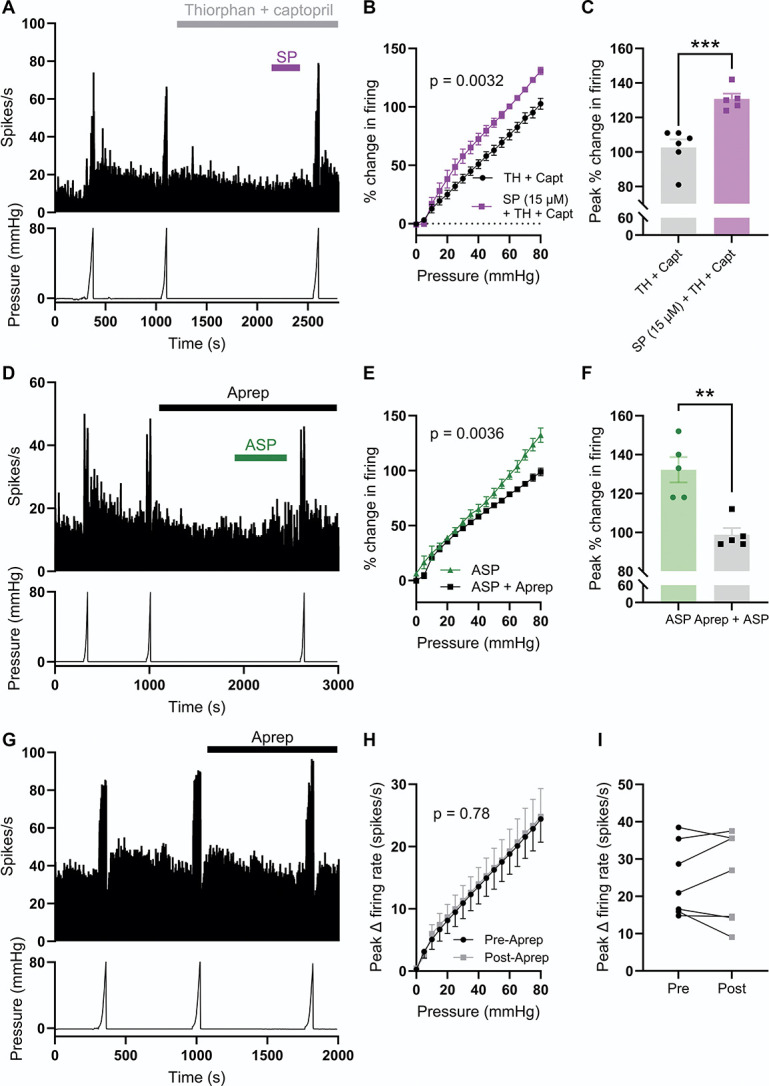
Substance P signalling is required for ASP7663-induced mechanical hypersensitivity. (A) Example recording showing afferent firing during 3 consecutive ramp distensions of the colon, with SP, thiorphan, and captopril applied between the second and third ramps. (B) Grouped data showing the percentage change in afferent firing during the third ramp distension after pretreatment with either thiorphan and captopril alone or thiorphan and captopril with 15 µM of SP. Two-way repeated measures ANOVA with Holm–Sidak post-hoc tests. (C) Grouped data showing the peak percentage change in afferent firing from the experiments shown in (A and B). Two-tailed unpaired *t* test. (D) Example recording showing afferent firing during 3 consecutive ramp distensions of the colon with aprepitant and ASP7663 applied between the second and third ramps. (E) Grouped data showing the percentage change in afferent firing during the third ramp distension after pretreatment with ASP7663 with or without aprepitant. Two-way repeated measures ANOVA with Holm–Sidak post-hoc tests. (F) Grouped data showing the peak percentage change in afferent firing from the experiments shown in (D and E). Two-tailed unpaired *t* test. (G) Example recording showing afferent firing during 3 ramp distensions with aprepitant alone applied between the second and third ramps. (H) Grouped data showing the change in afferent firing rate during the second (preaprepitant) and third (postaprepitant) ramp distensions. Two-way repeated measures ANOVA with Holm–Sidak post-hoc tests. (I) Grouped data showing the peak change in afferent firing rate during the second (preaprepitant) and third (postaprepitant) ramp distensions. Two-tailed paired *t* test. SP, substance P.

Building on the observation that SP mediates, at least in part, the activation of colonic afferents in response to the stimulation of TRPA1, we next sought to understand the contribution of SP to TRPA1 agonist–induced mechanical hypersensitivity. First, we demonstrated that application of 15 µM of SP, in the presence of thiorphan and captopril, evoked mechanical hypersensitivity comparable to that observed after ASP7663 application (Fig. 7A). Substance P augmented distension-evoked afferent firing (main effect of drug, F(1, 9) = 15.8, *P* = 0.0032, Fig. 7B). The peak change in distention-evoked afferent firing was 102.7 ± 4.7% in control conditions (N = 6) compared to 130.8 ± 3.1% after SP application (N = 5, *P* = 0.0009, Fig. 7C).

Furthermore, pretreatment with 10 µM of aprepitant ameliorated ASP7663-evoked mechanical hypersensitivity (main effect of drug, F(1, 8) = 16.5, *P* = 0.0036, Figs. 7D and E), reducing the peak change in distension-evoked afferent firing from 132.2 ± 6.5% (N = 5) to 94.0 ± 3.4% (N = 5, *P* = 0.0019, Fig. 7F). Treatment with aprepitant alone had no effect on distension-evoked firing (main effect of drug, F(1, 6) = 0.085, *P* = 0.78, Figs. 7G and H). There was no difference in peak distension–evoked afferent firing before and after the application of aprepitant (N = 7, *P* = 0.84, Fig. 7I). Treatment with SP, aprepitant and ASP7663, or aprepitant alone did not affect colonic compliance (Supplemental Figure 5, http://links.lww.com/PAIN/C131).

### 3.8. Cromolyn blocks transient receptor potential ankyrin 1–mediated substance P release and colonic contractility via GPR35

**Figure 8.**
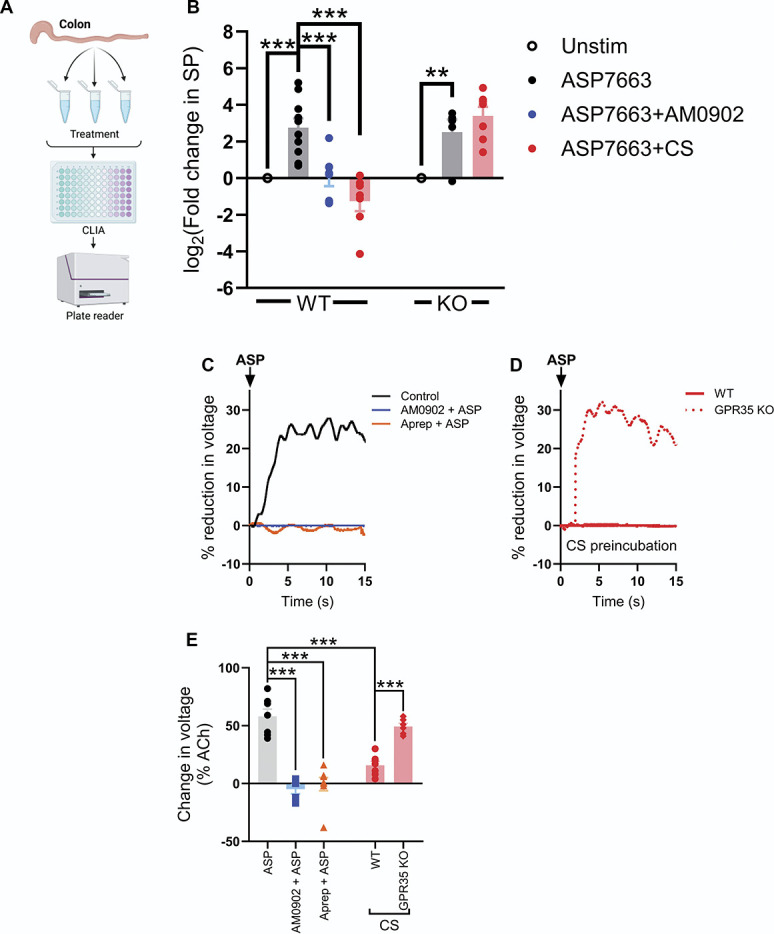
TRPA1 activation evokes SP release in the colon. (A) Schematic showing the experimental protocol for measuring SP release in the colon. (B) Grouped data showing the fold change in SP (relative to a tissue-matched unstimulated control experiment) after incubation of tissue with ASP7663 alone or in the presence of AM0902 or CS in wildtype and GPR35^−/−^ tissue. One-way ANOVA with Bonferroni post-hoc tests. (C) Example recordings showing the reduction in colon length by ASP7663 (shown as a reduction in voltage across a transducer attached to one end of the colon; voltages are shown relative to that evoked by 10 µM of ACh). The effect of ASP7663 was blocked by both AM0902 and aprepitant. (D) Example recordings showing the effect of CS pretreatment on ASP7663-induced colonic contraction in tissue from wildtype and GPR35^−/−^ animals. CS failed to inhibit ASP7663-induced contraction in tissue lacking GPR35. (E) Grouped data showing the peak percentage change in transducer voltage (relative to ACh) for the experiments shown in (C and D). One-way ANOVA with Bonferroni post-hoc tests. CS, cromolyn; SP, substance P; TRPA1, transient receptor potential ankyrin 1.

Having identified a role for SP in TRPA1-mediated activation of colonic afferents and mechanical hypersensitivity, we sought to confirm that TRPA1 activation evokes SP release in colonic tissue and whether this could be attenuated by agonist stimulation of GPR35. To do so, we first directly measured SP release from the colon in response to ASP7663 using a chemiluminescent immunoassay (CLIA, Fig. 8A, Table 1). Treatment of wildtype tissue with ASP7663 (30 µM) elicited a ∼10-fold increase in detected SP compared to unstimulated control tissue (N = 10 per group, 0.274 ± 0.102 pg/mg vs 2.403 ± 1.038 pg/mg, F(6, 47) = 17.2, *P* < 0.0001, Fig. 8B). This was blocked by 3 µM of AM0902 (0.277 ± 0.076 pg/mg, N = 8, *P* < 0.0001, Fig. 8B), confirming SP release was mediated by TRPA1 activation. ASP7663-evoked SP release was also abolished by pretreatment with 100 µM of CS (0.133 ± 0.038 pg/mg, N = 6, *P* < 0.0001, Fig. 8B).

**Table 1:** Substance P release evoked by activation of transient receptor potential ankyrin 1.

	GPR35^+/+^				GPR35^−/−^		
	Unstim	ASP7663	ASP7663+ AM0902	ASP7663+ CS	Unstim	ASP7663	ASP7663+ CS
Mean SP (pg/mg)	0.274	2.403	0.277	0.113	0.335	1.65	3.92
SEM (pg/mg)	0.102	1.038	0.076	0.038	0.097	1.15	1.47

Data show mean SP detected (pg SP/mg tissue) using a chemiluminescent immunoassay (analysis shown in Fig. 8B).

SP, Substance P.

By contrast, CS had no effect on the magnitude of SP release evoked by ASP7663 in tissue from GPR35^−/−^ mice. Treatment of tissue from GPR35^−/−^ mice with ASP7663 resulted in a robust (∼6-fold) increase in detected SP compared with the unstimulated control (*P* = 0.0034). Unlike tissue from wildtype mice, ASP7663-induced SP release in tissue from GPR35^−/−^ mice was unchanged in the presence (N = 7) or absence (N = 5) of CS (*P* > 0.99, Fig. 8B), indicating the loss of the inhibitory effect of CS.

Consistent with the findings from the SP release assay, the application of ASP7663 evoked a marked contraction of the mouse distal colon (N = 7, Fig. 8C), which was abolished by pretreatment with either AM0902 (3 µM, N = 5, F(4, 28) = 33.1, *P* < 0.0001, Fig. 8C) or aprepitant (10 µM, N = 8, *P* < 0.0001, Fig. 8C), consistent with the response being mediated by TRPA1-evoked SP release. Pretreatment with 100 µM of CS robustly inhibited ASP7663-evoked colonic contraction in tissue from wildtype mice (N = 7, *P* < 0.0001 compared to ASP7663 alone, Fig. 8D). CS had no effect on the contractile response to ASP7663 application in tissue from GPR35^−/−^ mice (N = 6, *P* > 0.99 compared to ASP7663 alone in wildtype tissue, Fig. 8D). The effect of CS on ASP7663-evoked colonic contraction was markedly different in tissue from wildtype and GPR35^−/−^ animals (*P* = 0.0003, Fig. 8E); the inhibitory effect of CS was lost in GPR35^−/−^ tissue.

### 3.9. Cromolyn blocks transient receptor potential ankyrin 1–mediated colonic afferent activation and mechanical hypersensitivity in tissue from female mice

**Figure 9.**
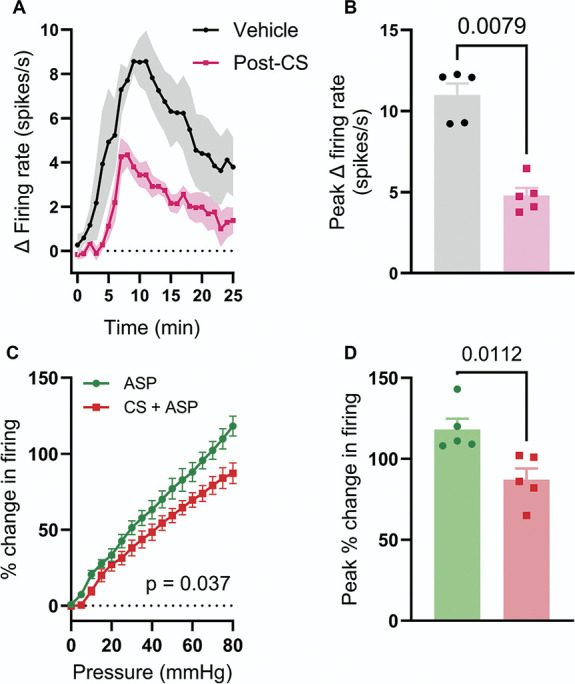
CS inhibits ASP7663-induced afferent activity and mechanical hypersensitivity in tissue from female mice. (A) Grouped data showing the change in afferent firing after the application of ASP7663 (30 µM) to tissue preincubated with vehicle (black) or CS (100 µM, pink). Solid points show the mean change in afferent firing; shaded regions show SEM. (B) Grouped data showing the change in afferent firing evoked by ASP7663 in vehicle- and CS-treated tissue. Data analysed using a two-tailed Mann–Whitney *U* test. (C) Grouped data showing the peak change in afferent firing during the third (post-ASP7663) ramp distension in tissue treated with ASP7663 with or without CS. Two-way repeated measures ANOVA. (D) Grouped data showing the peak percentage change in afferent firing during the third (post-ASP7333) ramp distension in tissue treated with ASP7663 alone (green) or ASP7663 and CS (red). Two-tailed unpaired *t* test. CS, cromolyn.

To confirm that the inhibitory effect of CS on TRPA1-mediated colonic afferent activation translated from male to female animals, tissue from female C57BL/6 mice was treated with ASP7663 (30 µM) with and without preincubation with CS (100 µM). CS preincubation resulted in a significant reduction in the afferent response to ASP7663 (4.8 ± 0.5 spikes/s, N = 5) compared with vehicle-treated tissue (11.0 ± 0.7 spikes/s, N = 5, *P* = 0.0079) (Figs. 9A and B).

Treatment of tissue from female mice with ASP7663 induced afferent mechanical hypersensitivity (main effect of drug, F(1,4) = 11.2, *P* = 0.029) with no change in colonic compliance (Supplemental Figure 6A, http://links.lww.com/PAIN/C131). What's more, preincubation of tissue from female mice with CS (100 µM) attenuated ASP7663-induced mechanical hypersensitivity (main effect of drug, F(1, 8) = 6.23, *P* = 0.037, Fig. 9C). CS suppressed ASP7663-induced sensitisation of the peak change in distension-evoked afferent firing (ASP7663 alone: 118.2 ± 6.6%; ASP7663 + CS: 87.2 ± 6.8%, *P* = 0.011, Fig. 9D). CS had no effect on colonic compliance in tissue from female animals (Supplemental Figure 6B, http://links.lww.com/PAIN/C131). These data verify that the effects of CS are similar in male and female mice.

### 3.10. Substance P–dependent afferent activity evoked by prolonged distension of the colon

**Figure 10.**
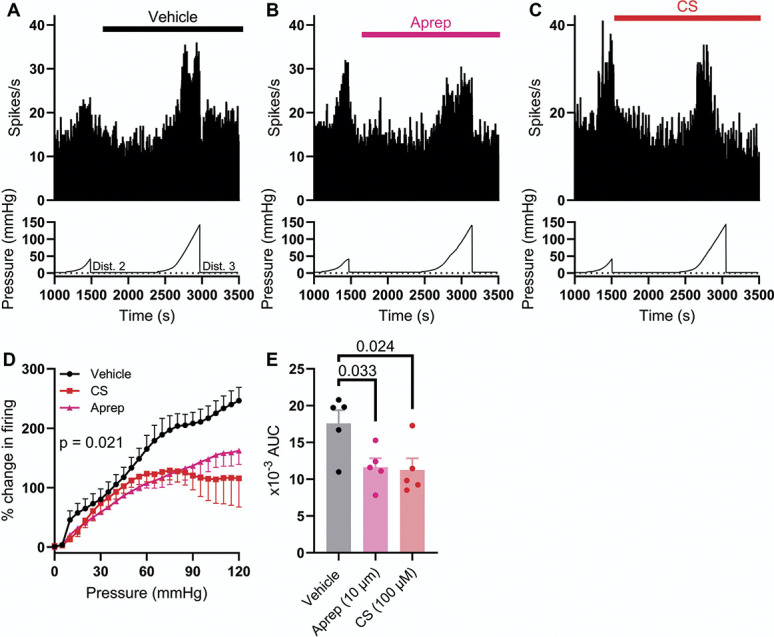
Prolonged colonic distension evoked SP-dependent colonic afferent activity. (A) Example rate histogram (top) and colonic luminal pressure trace (bottom) showing the afferent response to slow ramp distension to 40 mm Hg (Dist. 2) and 120 mm Hg (Dist. 3) in the presence of vehicle (1:1000 DMSO). (B) Example rate histogram (top) and colonic luminal pressure trace (bottom) showing the afferent response to slow ramp distension to 40 mm Hg (Dist. 2) and 120 mm Hg (Dist. 3) in the presence of aprepitant (10 µM). (C) Example rate histogram (top) and colonic luminal pressure trace (bottom) showing the afferent response to slow ramp distension to 40 mm Hg (Dist. 2) and 120 mm Hg (Dist. 3) in the presence of CS (100 µM). (D) Grouped data showing the percentage change in afferent activity during the third ramp distension (to 120 mm Hg) in the presence of vehicle (black), aprepitant (pink), and CS (red). Two-way repeated measures ANOVA with Holm–Sidak post-hoc tests. (E) Grouped data showing the area under the pressure-response curves (AUC) in (D). One-way ANOVA with Dunnett post-hoc tests. CS, cromolyn; SP, substance P.

Given the mechanical sensitivity of TRPA1, we hypothesised that prolonged, high-pressure distension of the colon would lead to SP-dependent afferent firing downstream of sustained TRPA1 activation and antidromic SP release. To test this hypothesis, the colon was distended from 0 to 120 mm Hg at a reduced rate of 1 mL/h (∼600 seconds, Fig. 10A), rather than distending from 0 to 80 mm Hg at a rate of 6 mL/h (∼100 seconds). Before this prolonged distension, the colon was twice distended from 0 to 40 mm Hg at the same rate to ensure stable pressure-evoked afferent responses and such that afferent firing during the third distension (to 120 mm Hg) could be normalised against the second distention (to 40 mm Hg, Fig. 10A). Afferent activity during the third distension was measured after preincubation with vehicle (0.1% DMSO, Fig. 10A), aprepitant (10 µM, Fig. 10B), and CS (100 µM, Fig. 10C).

Aprepitant suppressed pressure-evoked afferent activity during prolonged distension to 120 mm Hg (main effect of drug, F(2,12) = 5.40, *P* = 0.021, Fig. 10D). In vehicle control experiments, peak afferent activity during the third distension (at 120 mm Hg) was 246.4 ± 22.5% (N = 5) of that of the peak during the second distension (at 40 mm Hg, Fig. 10D). However, after incubation with aprepitant, peak afferent activity was reduced to 162.2 ± 22.9% (N = 5, *P* = 0.0048, Fig. 10D). The area under the pressure-activity curve (AUC) was also reduced from 17.6 × 10^3^ ± 1.8 × 10^3^ AU (vehicle) to 11.6 × 10^3^ ± 1.2 × 10^3^ AU (aprepitant, F(2,12) = 5.31, *P* = 0.033, Fig. 10E). These data indicate that pressure-evoked firing in this prolonged (∼600 seconds) distension protocol is, at least in part, dependent on SP signalling, in line with antidromic SP release after mechanical stimulation of TRPA1.

Preincubation with CS attenuated pressure-evoked afferent firing compared to the vehicle control (main effect of drug, F(2,12) = 5.40, *P* = 0.021, Fig. 10D). Peak afferent activity (at 120 mm Hg) in the presence of CS was 115.8 ± 48.4% (N = 5, *P* < 0.0001, Fig. 10D). The AUC was similarly reduced by CS (11.3 × 10^3^ ± 1.6 × 10^3^ AU, *P* = 0.024, Fig. 10E). This observation is in line with CS inhibiting SP release, thereby suppressing SP-dependent afferent activity in this protocol. These observations are at odds with those made using brief (∼100 seconds) ramp distensions, wherein pressure-evoked afferent activity was insensitive to inhibition by CS (Fig. 4) and aprepitant (Fig. 7). Colonic compliance during this prolonged distension protocol was no different in vehicle-, aprepitant-, and CS-treated preparations (Supplemental Figure 7, http://links.lww.com/PAIN/C131).

## 4. Discussion

The development of nonopioid analgesics for the treatment of chronic abdominal pain is a pressing area of unmet clinical need. To address this, we examined the distribution of G_i/o_-coupled receptors in colonic sensory neurons to identify drug targets, which have the potential to inhibit nociceptor activation but lack the abuse liability, sedation, and gut-related side effects associated with agonists of opioid, cannabinoid, and GABA_B_ receptors.^57^ This led to the identification of several G_i/o_-coupled GPCRs as putative visceral analgesic drug target because of their marked coexpression with TRPA1, a mediator of noxious mechanosensitivity in the bowel.^12^ We examined the antinociceptive effects of GPR35 stimulation because of the commercial availability of GPR35 agonists, such as CS and Zap, and GPR35^−/−^ mice.

Consistent with our in silico docking studies, which identified binding sites for CS and Zap on the GPR35 receptor, we demonstrated that the antinociceptive effects of these drugs on TRPA1-mediated colonic nociceptor activation and mechanosensitisation were dependent upon GPR35. Further work showed that these antinociceptive effects occurred through the inhibition of SP release and, in doing so, revealed both the pronociceptive effect of SP on colonic afferent activity and the contribution of SP to TRPA1-mediated colonic nociceptor activation. In addition, our data identify GPR35, and its inhibition of SP-mediated colonic contractility and nociceptor firing, as a putative mechanism of action for the improvement in symptoms reported with CS in people with IBS.^17,23,58,59^ These findings highlight the utility of GPR35 agonists for the treatment of abdominal pain in IBS, as well as other GI diseases, such as IBD.

The findings from our study are strongly supported by the literature on transcript expression in sensory neurons in the DRG. Previous reports show marked expression of GPR35 in sensory neurons,^21,34,47,51,65^ as well as its coexpression with TRPA1 and SP.^34^ Importantly, this coexpression is conserved in human DRG in which GPR35 is highly expressed in nociceptor populations, including those with enriched expression of TRPA1.^62^ Although not reported in the current study, GPR35 also shows marked coexpression with other channels and receptors responsible for nociceptive signalling in colonic afferents, such as TRPV1, Na_V_1.8, tropomyosin receptor kinase A (TrkA), histamine H_1_ receptors, and 5-HT_3_ receptors.^21,26,29,34,41,51^ As such, we speculate that the inhibitory effect of GPR35 stimulation on TRPA1-mediated colonic afferent activation and mechanical hypersensitivity may be replicated across a broader range of noxious and disease-derived stimuli such as acidity, NGF, histamine, and 5-HT, although further investigation is required to resolve this.

In the present study, we used TRPA1-induced afferent activity and hypersensitivity to study the antinociceptive effects of GPR35 on colonic afferent activation because of its established role in noxious colonic mechanosensation.^12,45,64,68^ In addition, TRPA1 is a downstream effector for the development of visceral mechanical hypersensitivity in response to a broad range of algogenic stimuli implicated in IBS and IBD.^16^ These include inflammatory mediators such as bradykinin,^2,8^ histamine,^1^ prostaglandins (PGE_2_),^22^ interleukins (IL-1β) and TNFα,^6,37^ as well as inflammatory supernatants generated from animal models of visceral hypersensitivity, and IBS and IBD biopsy samples.^37,67^ We confirmed the pronociceptive potential of TRPA1 by showing that pretreatment with a selective TRPA1 antagonist, AM0902, inhibited the colonic afferent response to luminal distension at noxious distending pressures and demonstrated that pretreatment with a TRPA1 agonist, ASP7663, produced mechanical hypersensitivity.

Interestingly, the effect of GPR35 activation showed evidence of stimulus selectivity, inhibiting TRPA1-mediated colonic afferent activation and mechanosensitisation but not the response to luminal distension at noxious pressures known to be dependent on TRPA1. Further study revealed that this was because of inhibition of SP released after agonist stimulation of TRPA1, which contributed significantly to ASP7663-induced colonic afferent activation and mechanosensitisation as revealed by the inhibition of these effects by aprepitant. However, aprepitant did not suppress the basal afferent response to colonic distension, indicating that SP release does not contribute to distension-evoked afferent firing. This is most likely a consequence of the duration of afferent and TRPA1 channel activity during colonic distension being insufficient to trigger substantive antidromic SP release from nerve terminals. Sustained agonist-induced activity of TRPA1 is seemingly better suited to evoking SP release.^27,49,63^ We further tested this by probing the effect of prolonged, high-pressure distension of the colon (luminal pressure raised to 120 mm Hg over ∼600 seconds), as it has previously been shown that colonic distension can evoke SP release.^56^ Pressure-evoked afferent activity in this prolonged distension protocol was inhibited by aprepitant, indicating the antidromic release of SP and subsequent afferent stimulation through NK1R. In line with our observation that CS inhibits SP release, we also found that CS suppressed afferent activity in this prolonged distension protocol. Prolonged distension of the colon is an important pathophysiological event in GI disease (eg, bloating and constipation in IBS) and, given our observations, GPR35 agonists may be effective in relieving the associated pain by suppressing colonic afferent activity.

Building on this observation, it may be expected that GPR35 agonists would have the desirable effect of preventing the prolonged activation of nociceptors associated with visceral hypersensitivity and chronic pain in diseases such as IBS and IBD without blocking acute abdominal pain arising in response to potentially life-threatening obstruction of the bowel. Consistent with this hypothesis, CS has previously been reported to reduce symptom severity in IBS patients,^23,58,59^ an effect our findings suggest is mediated by GPR35 receptor activation.

In addition to inhibiting TRPA1-mediated colonic afferent activation, mechanical hypersensitivity, SP release, and colonic contraction (detailed in the present study), CS has also been reported to have several desirable actions on immune cell function.^54^ Most notably, CS acts as a mast cell stabilizer, which is also likely to inhibit colonic afferent activation and pain in disease states such as IBS and IBD.^3,10,42,58,59^ Our data suggest that mast cells are not responsible for the inhibitory effects of CS observed in the current study as responses were replicated with Zap, which is not a mast cell stabiliser.^17^ Furthermore, experiments were also performed using tissue from healthy mice in which colonic mast cell infiltration is typically low and, as such, unlikely to contribute significantly to changes in colonic afferent activity. This was confirmed by the inability of compound 48/80 to stimulate colonic afferent firing, consistent with previous findings from other groups.^20^ Nevertheless, the ability of CS to suppress colonic afferent activity, colonic contractility, and the release of proinflammatory and algogenic mediators from mast cells is likely to result in greater symptom improvement because of the targeting of multiple aspects of disease pathology.

## 5. Conclusions

The findings from our study highlight the potential of GPR35 agonists to provide non–opioid-based analgesia in GI diseases, such as IBS and IBD, because of their ability to prevent neurogenic colonic afferent activation, mechanosensitisation, and colonic contractility through the inhibition of SP release. These findings support the further development of GPR35 agonists for the treatment of GI disorders.

## Conflict of interest statement

Dr Rie Suzuki and Dr Alastair Brown are employed by Sosei-Heptares. Dr David Bulmer receives research funding from Sosei-Heptares and GlaxoSmithKline.

## Appendix A. Supplemental digital content

Supplemental digital content associated with this article can be found online at http://links.lww.com/PAIN/C131.
