# Supplementary material for: GPR35 agonists inhibit TRPA1-mediated colonic nociception through suppression of substance P release
Source: Pain. 2024 Oct 3;166(3):596–613. doi: 10.1097/j.pain.0000000000003399 (PMC11808708; doi:10.1097/j.pain.0000000000003399)
Supplement: Supplementary file 1 [file jop-166-596-s001.pdf]

Supplemental Figure 1: ASP7663 stimulates colonic afferent firing through TRPA1 activation.

(A) Grouped data showing the mean change in afferent firing following the application 3, 9 or 30 µM ASP7663. Symbols show the mean firing rate and error bars show the standard error.

(B) Grouped data showing the mean peak change in afferent firing rate following the application of 9 µM ASP7663 to tissue in control conditions or pre-incubated with the TRPA1 antagonist AM0902 (1 µM). Two-tailed unpaired t-test.

(C) Grouped data showing the mean change in afferent firing following the first and second application of ASP7663.

(D) Grouped data showing the mean peak change in afferent firing following the first and second application of ASP7663. Two-tailed ratio-paired t-test.

Supplemental Figure 2: Off-target effects of CS and Zap are unlikely to mediate their inhibition of ASP7663-evoked afferent firing.

(A) Example rate histogram showing the afferent firing rate before and during the application of Compound 48/80.

(B) Grouped data showing the mean change in afferent firing following the application of ASP7663 (30 µM) to control tissue (black) and tissue pre-incubated with IBMX (50 µM, orange) or sildenafil (1 µM, pink).

(C) Grouped data showing the area under the afferent response curves in (B). One-way ANOVA with Tukey's post-hoc tests.

Supplemental Figure 3: Colonic compliance is not affected by inhibition or activation of TRPA1.

(A) Grouped data showing the peak change in afferent firing for each of five consecutive ramp distensions to 80 mmHg. Peak afferent firing during ramps 2 and 3 is indistinguishable ($p = 0.87$). One-way repeated measures ANOVA (main effect of distension, $p = 0.0071$; ramp 1 vs ramp 5, $p = 0.021$; ramp 3 vs ramp 5, $p = 0.020$).

(B) Grouped data showing the volume needed to raise the luminal pressure to 80 mmHg before and after AM0902. Two-way repeated measures ANOVA (main effect of drug, $p = 0.90$).

(C) Grouped data showing the volume needed to raise the luminal pressure to 80 mmHg before and after CS application. Two-way repeated measures ANOVA (main effect of drug, $p = 0.69$).

(D) Grouped data showing the volume needed to raise the luminal pressure to 80 mmHg before and after Zap application. Two-way repeated measures ANOVA (main effect of drug, $p = 0.69$).

Supplemental Figure 4: Stimulation of GPR35 does not change the compliance of the colon.

(A) (Left) Grouped data showing the volume needed to raise the luminal pressure to 80 mmHg before and after ASP7663 application to wildtype tissue. Two-way repeated measures ANOVA (main effect of drug, $p = 0.78$). (Right) Grouped data showing the volume needed to raise the luminal pressure to 80 mmHg before and after ASP7663 application to GPR35$^{-/-}$ tissue. Two-way repeated measures ANOVA (main effect of drug, $p = 0.92$).

(B) (Left) Grouped data showing the volume needed to raise the luminal pressure to 80 mmHg before and after CS and ASP7663 application to wildtype tissue. Two-way repeated measures ANOVA (main effect of drug, $p = 0.52$). (Right) Grouped data showing the volume needed to raise the luminal pressure to 80 mmHg before and after CS and ASP7663 application to GPR35$^{-/-}$ tissue. Two-way repeated measures ANOVA (main effect of drug, $p > 0.99$).

(C) Grouped data showing the volume needed to raise the luminal pressure to 80 mmHg before and after Zap and ASP7663 application to wildtype tissue. Two-way repeated measures ANOVA (main effect of drug, $p = 0.59$).

Supplemental Figure 5: NK1 receptor inhibition does not affect colonic compliance.

(A) Grouped data showing the volume needed to raise the luminal pressure to 80 mmHg before and after SP application to wildtype tissue. Two-way repeated measures ANOVA (main effect of drug, $p = 0.75$).

(B) Grouped data showing the volume needed to raise the luminal pressure to 80 mmHg before and after aprepitant and ASP7663 application to wildtype tissue. Two-way repeated measures ANOVA (main effect of drug, $p = 0.61$).

(C) Grouped data showing the volume needed to raise the luminal pressure to 80 mmHg before and after aprepitant application to wildtype tissue. Two-way repeated measures ANOVA (main effect of drug, $p = 0.77$).

Supplemental Figure 6: Lack of effect of ASP7663 and CS on colonic compliance in tissue from female mice.

(A) Grouped data showing the volume needed to raise luminal pressure to 80 mmHg before and after ASP7663 application in tissue from female animals. Two-way repeated measures ANOVA (main effect of drug, $p = 0.23$).

(B) Grouped data showing the volume needed to raise luminal pressure to 80 mmHg before and after CS and ASP7663 application in tissue from female animals. Two-way repeated measures ANOVA (main effect of drug, $p = 0.99$).

Supplemental Figure 7: Neither NK1 stimulation, nor GPR35 inhibition, affects colonic compliance during prolonged ramp distension to 120 mmHg.

Grouped data showing the volume needed to raise luminal pressure to 120 mmHg in the presence of vehicle (1:1000 DMSO), aprepitant and CS in wildtype tissue. Two-way repeated measures ANOVA (main effect of drug, p = 0.).

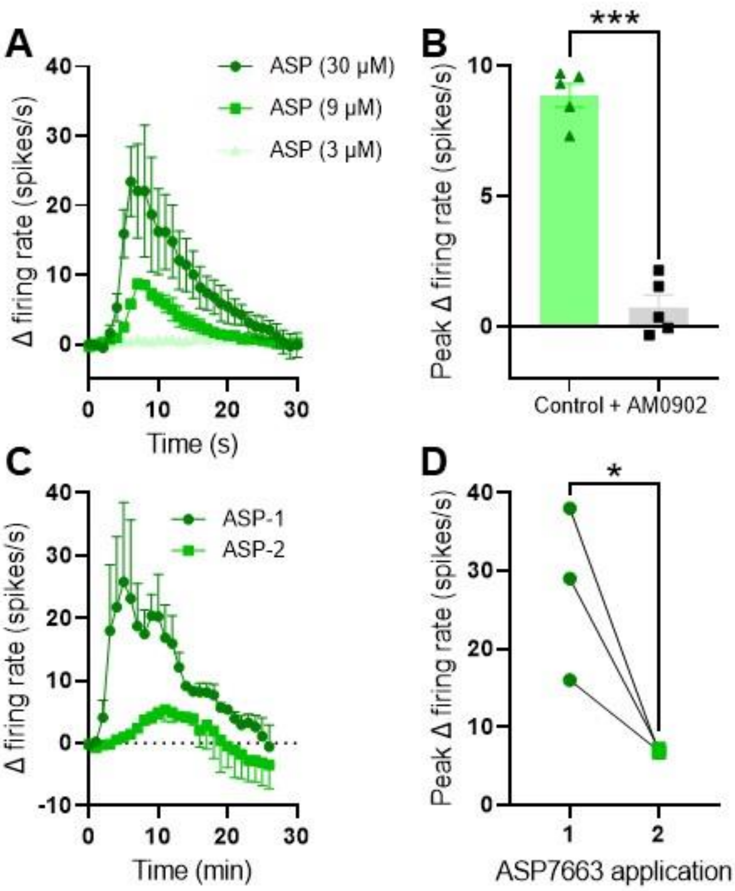

Supplemental Figure 1

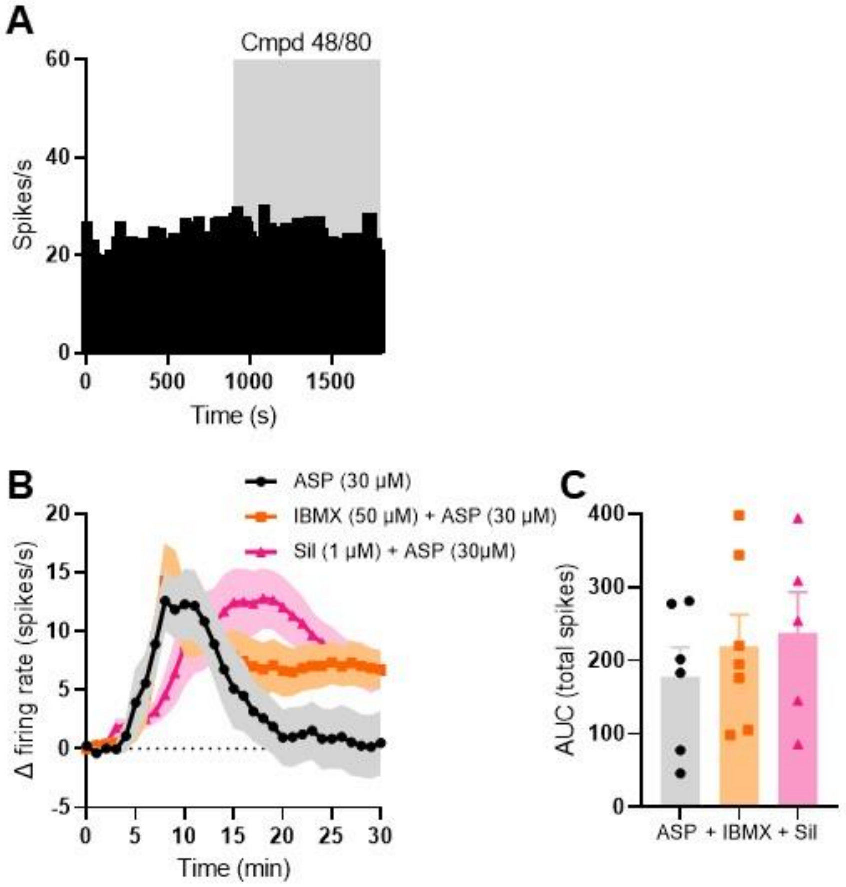

Supplemental Figure 2

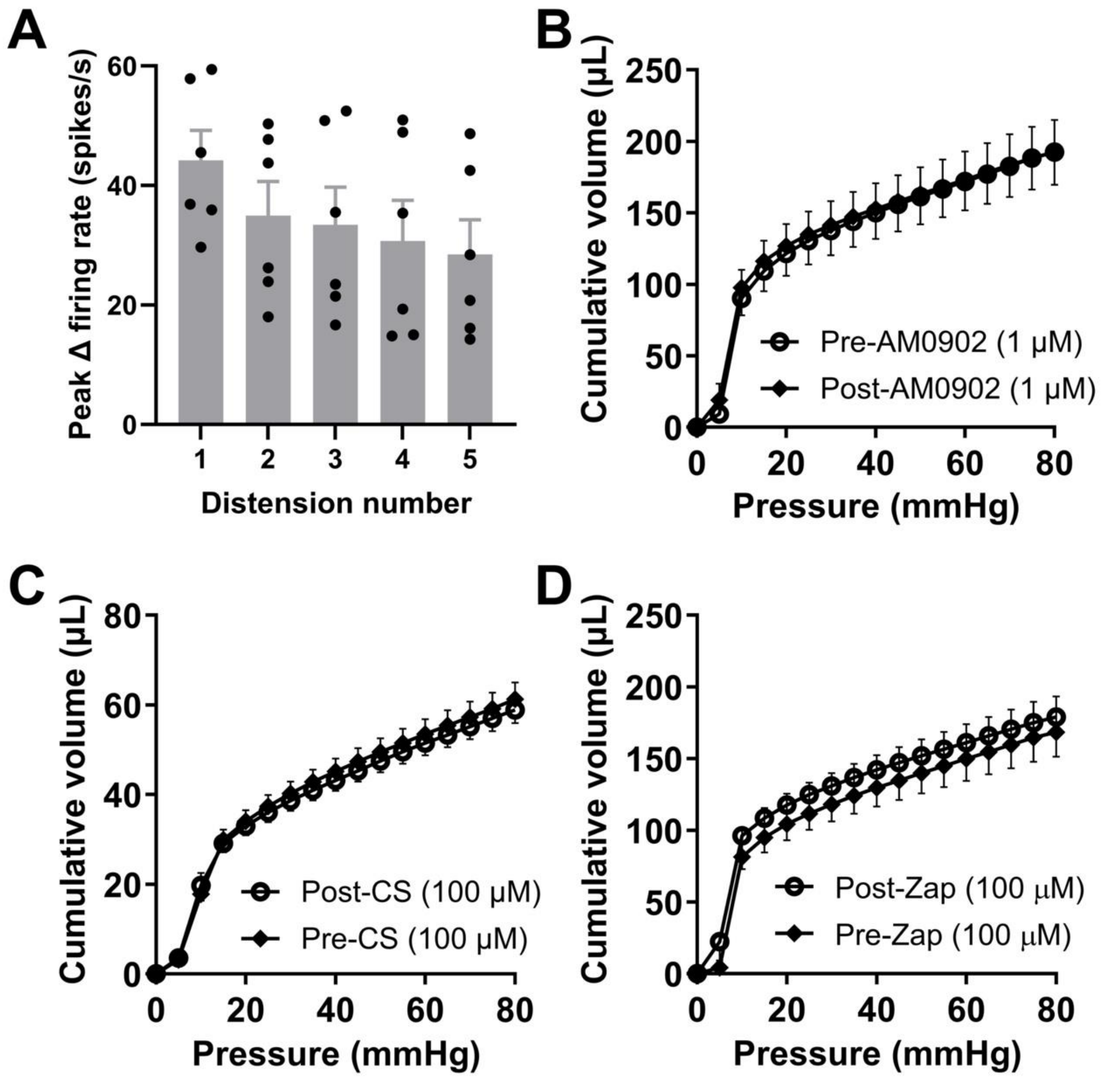

Supplemental Figure 3

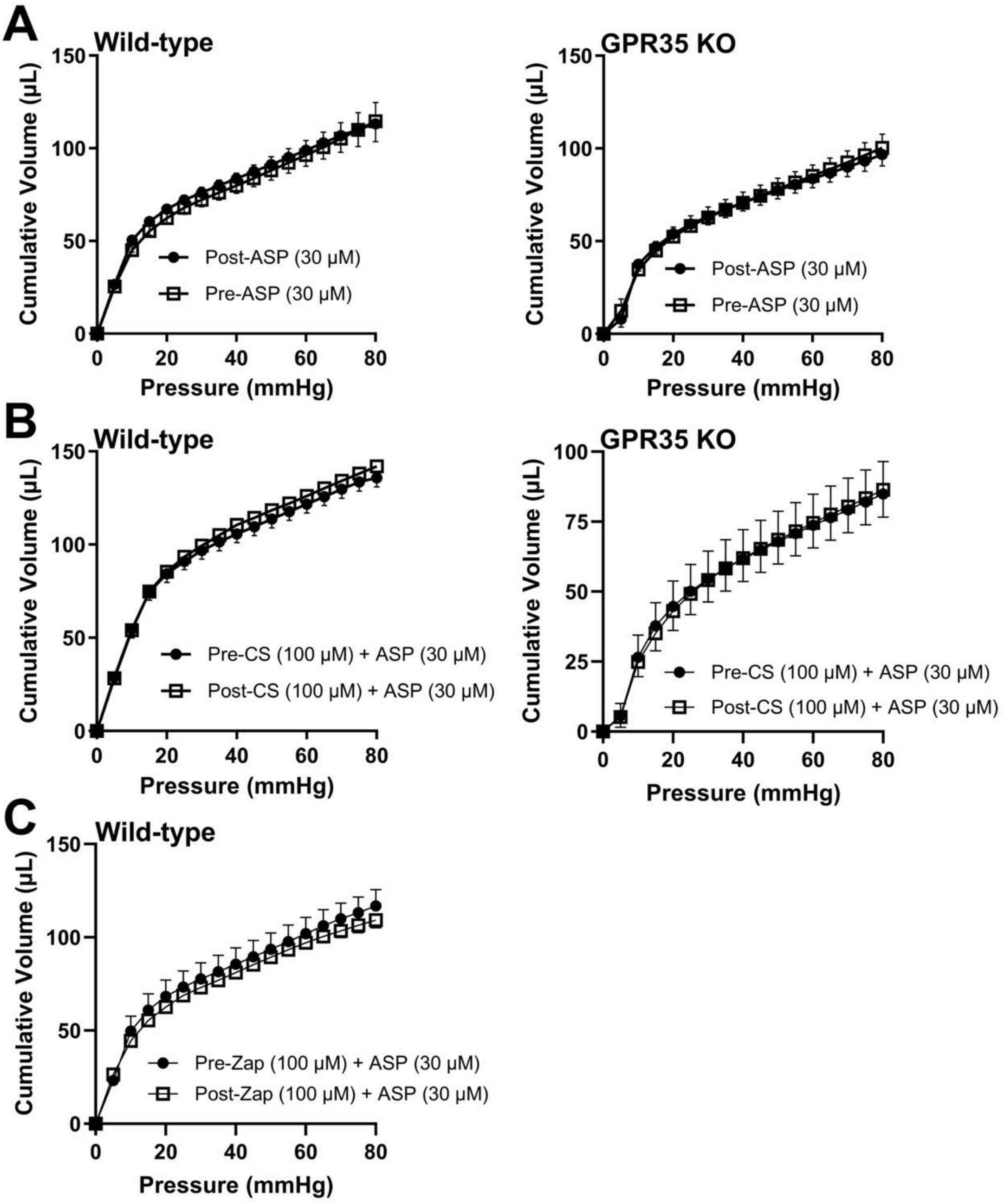

Supplemental Figure 4

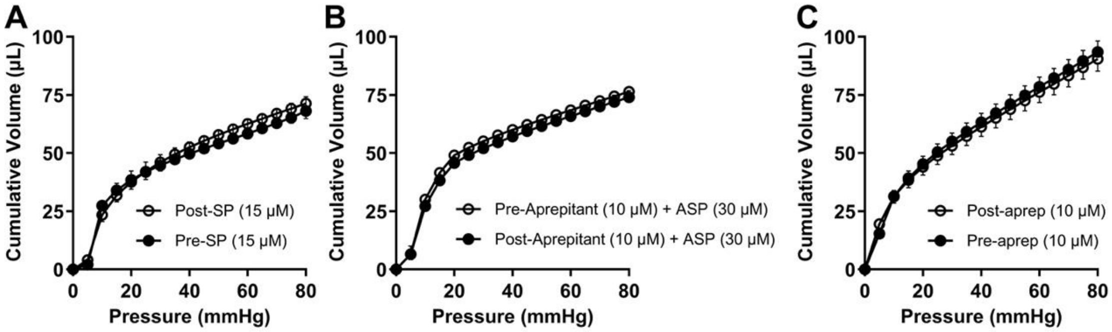

Supplemental Figure 5

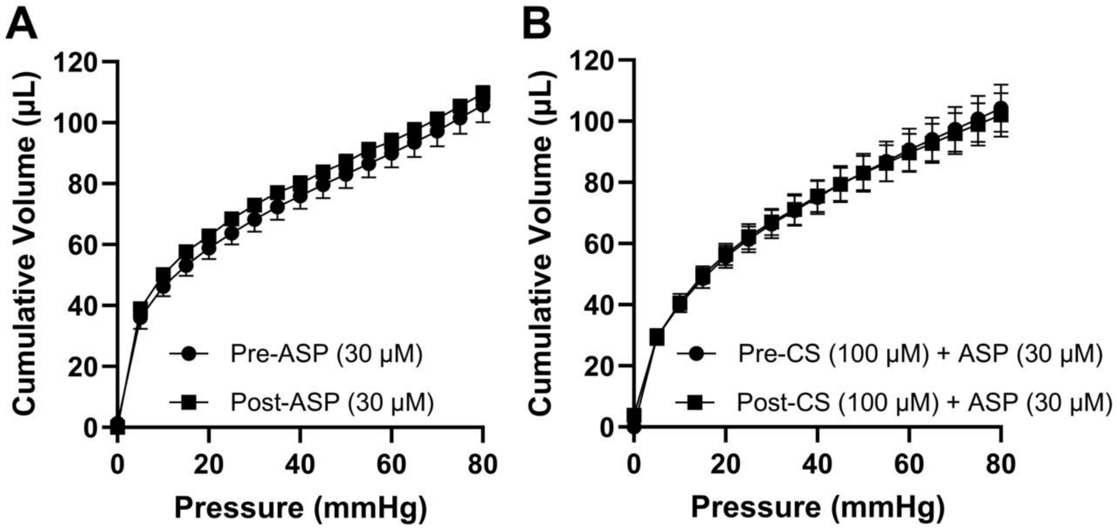

Supplemental Figure 6

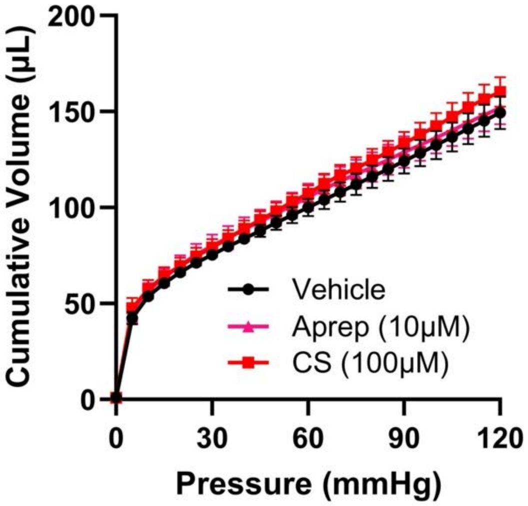

Supplemental Figure 7
