# Supplementary material for: GPR35 agonists inhibit TRPA1-mediated colonic nociception through suppression of substance P release
Source: Pain. 2024 Oct 3;166(3):596–613. doi: 10.1097/j.pain.0000000000003399 (PMC11808708; doi:10.1097/j.pain.0000000000003399)
Supplement: Supplementary file 2 [file jop-166-596-s002.pdf]

Re: URGENT: License to Publish request for PAIN-D-24-00185R1

David Bulmer <dcb53@cam.ac.uk>
Tue 7/30/2024 3:48 AM
To:Pain J <PainJ@iasp-pain.org>

Hi

I complete the section – but I now notice that the selection – which should be Research Councils UK (RCUK)
Has defaulted back to the original please select… text

I can change this online but when I download the form to sign it defaults by to the original, please select option.

Could you move forward/change with this email confirmation the option is RCUK?
I can't change this and sign on the downloaded form

Best wishes

David

Dr David Bulmer
Associate Professor
Part II Pharmacology Course Organiser
Department of Pharmacology
University of Cambridge

Bye-Fellow Wolfson College

Honorary Senior Lecturer
Barts and the London School of Medicine and Dentistry
Queen Mary University of London

From: Pain J <PainJ@iasp-pain.org>
Date: Monday, 29 July 2024 at 16:59
To: David Bulmer <dcb53@cam.ac.uk>
Subject: Re: URGENT: License to Publish request for PAIN-D-24-00185R1

Dr. Bulmer,

Please indicate which open access license you would like to publish under by indicating options, 1, 2, or 3.

Thank you and please let me know if you have any questions or concerns.

Best,

Renee Chasse | Publications Manager

International Association for the Study of Pain

1510 H Street NW, Suite 600 | Washington, DC 20005 | USA

www.painjournalonline.com | iasp-pain.org

Working Together for Pain Relief Throughout the World

From: David Bulmer <dcb53@cam.ac.uk>
Sent: Friday, July 26, 2024 4:58 PM
To: Pain J <PainJ@iasp-pain.org>
Subject: Re: URGENT: License to Publish request for PAIN-D-24-00185R1

PLEASE FIND ATTACHED

Dr David Bulmer
Associate Professor
Part II Pharmacology Course Organiser
Department of Pharmacology
University of Cambridge

Bye-Fellow Wolfson College

Honorary Senior Lecturer
Barts and the London School of Medicine and Dentistry
Queen Mary University of London

From: em.pain.0.8cfab8.1eb739cd@editorialmanager.com <em.pain.0.8cfab8.1eb739cd@editorialmanager.com> on behalf of Pain <em@editorialmanager.com>
Date: Friday, 26 July 2024 at 17:34
To: David Bulmer <dcb53@cam.ac.uk>
Subject: URGENT: License to Publish request for PAIN-D-24-00185R1

ID: PAIN-D-24-00185R1
Title: GPR35 agonists inhibit TRPA1 mediated colonic nociception through suppression of Substance P release
Format: Research Paper
Authors: Rohit A Gupta; James P Higham; Abigail Pearce; Paulina Urriola-Muñoz; Katie H Barker; Luke Paine; Joshua Ghoorarooo; Tim Raine; James RF Hockley; Taufiq Rahman; Ewan StJohn Smith; Alastair JH Brown; Graham Ladds; Rie Suzuki; David C Bulmer

Dear Dr. Bulmer,

Because you indicated at the revision stage that you would like your submission to be made Open Access, we require a completed License to Publish (LTP) form before your manuscript can be transmitted to production. Please download the form from http://links.lww.com/LWW-ES/A49, sign it, and email the completed form to the journal office. Note that your submission will remain on hold until we receive the completed form.

Please let us know if you have any questions, and we look forward to hearing from you soon.

Best wishes,

Editorial Office, *PAIN*
painj@iasp-pain.org

In compliance with data protection regulations, you may request that we remove your personal registration details at any time. (Remove my information/details). Please contact the publication office if you have any questions.
